# Chicken IDO2 and TDO2: Biochemical Characteristics and Regulatory Roles in Avian Tryptophan Metabolism and Inflammatory Response

**DOI:** 10.3390/ani16182874

**Published:** 2026-09-12

**Authors:** Wanli Li, Chen Zhang, Pinhui Wu, Kangfei Feng, Guozhi Zhang, Lin Yuan, Wei Jin, Bingxun Wang, Shengli Li, Wei Liu, Wenqing Li

**Affiliations:** 1 Institute of Animal Husbandry, Henan Academy of Agricultural Sciences, Zhengzhou 450002, China; liwanli@hnagri.org.cn (W.L.); zhangchen19940205@163.com (C.Z.); yuanlin315@126.com (L.Y.); rhettbutler@126.com (W.J.); wbx13298185299@126.com (B.W.); aabb26@126.com (S.L.); 2 The Shennong Laboratory, Zhengzhou 450002, China; 3 Henan Key Laboratory of Farm Animal Breeding and Nutritional Regulation, Zhengzhou 450002, China; 4 College of Life Science, Henan Agricultural University, Zhengzhou 450046, China; phwu2022@163.com (P.W.); 18836905473@163.com (K.F.); guozhi_zhang@126.com (G.Z.); liuv@henau.edu.cn (W.L.)

**Keywords:** avian biochemistry, tryptophan metabolism, IDO2, TDO2, inflammatory response

## Abstract

Indoleamine 2,3-dioxygenase 2 (IDO2) and tryptophan 2,3-dioxygenase (TDO2) are rate-limiting enzymes of the kynurenine pathway, well-characterized in mammals. Unlike all other vertebrates, chickens lack the IDO1 gene and exclusively rely on IDO2/TDO2 for tryptophan (Trp) catabolism; however, their biochemical features and physiological regulatory functions remain poorly defined. Here, we integrated dietary Trp intervention, multi-omics profiling, recombinant enzyme kinetics, and in vitro/inflammatory animal models to characterize chicken IDO2 and TDO2. Supplementary dietary Trp maintained circulating Trp homeostasis while activating hepatic kynurenine metabolism being associated with reduced PPAR signaling and altered lipid and energy balance. Hepatic combined IDO2/TDO2 catalytic activity increased markedly with Trp supplementation; this increase was accompanied by only modest transcriptional upregulation of kynurenine pathway genes (including IDO2, TDO2, and KYNU) and no major changes in their protein abundance, indicating post-translational functional modulation. Recombinant enzyme assays demonstrated optimal activity at chicken physiological pH of 7.2 and temperature of 42 °C; chicken IDO2 displayed measurable catalytic efficiency, which was lower than that of chicken TDO2, whereas its human ortholog showed no detectable activity under the same conditions. Furthermore, LPS and APEC infection significantly suppressed the combined total IDO2/TDO2 activity during inflammatory stress, revealing an avian-specific metabolic–immune regulatory pattern. This work defines the unique biochemical and physiological functions of avian IDO2 and TDO2, offering mechanistic references for poultry nutritional regulation and disease control.

## Simple Summary

Indoleamine 2,3-dioxygenase 2 (IDO2) and tryptophan 2,3-dioxygenase (TDO2) serve as rate-limiting enzymes for the kynurenine pathway. Distinct from mammals, chickens lack the IDO1 gene and rely exclusively on IDO2 and TDO2 for tryptophan catabolism. Supplemental dietary tryptophan increases the combined hepatic total catalytic activity of IDO2/TDO2, accompanied by only modest transcriptional changes and no major changes at the protein level, and is associated with reduced PPAR signaling and altered lipid–energy balance. Recombinant enzyme assays demonstrated that chicken IDO2 and TDO2 achieve optimal catalytic performance at avian physiological pH of 7.2 and body temperature of 42 °C; chicken IDO2 possesses measurable catalytic efficiency, which is lower than that of chicken TDO2, whereas its human ortholog showed no detectable activity under the same conditions. Inflammatory stimuli including LPS and APEC infection significantly suppressed the combined hepatic IDO2/TDO2 dioxygenase activity, showing an avian-specific metabolic–immune regulatory feature. This study characterizes avian IDO2 and TDO2 biochemically and functionally, and provides theoretical basis for poultry nutritional regulation and disease prevention.

## 1. Introduction

Tryptophan (Trp) is an essential amino acid in animals; it is a raw material for protein synthesis and a precursor of biologically active molecules (such as serotonin, melatonin, and nicotinic acid). The main metabolic pathway is the kynurenine pathway (KP). The initial and rate-limiting steps are catalyzed by three enzymes: indoleamine 2,3-dioxygenase 1 (IDO1), indoleamine 2,3-dioxygenase 2 (IDO2), and tryptophan 2,3-dioxygenase (TDO2) [1,2]. IDO1 is expressed in extrahepatic tissues, particularly in immune cells and at sites of inflammation. Its expression is induced by proinflammatory cytokines, such as interferon-gamma (IFN-γ), and mediates the regulation of the local immune microenvironment [3,4]. IDO2 is a homolog of IDO1. Although it catalyzes the same reaction, its enzymatic activity is lower than that of IDO1 in mammals (especially humans and mice) [5]. Its exact function and regulatory mechanisms remain controversial, with studies suggesting that it may play a role in non-enzymatic activity [5,6]. TDO2 is primarily expressed in the liver and maintains systemic Trp levels by degrading approximately 90% of dietary Trp [7,8]. Under normal conditions, TDO2 contributes more to systemic Trp degradation than IDO1 [9]. These enzymes construct a metabolism–immune regulatory network by consuming Trp and producing kynurenine metabolites (such as kynurenine and kynurenic acid) [10]. In mammals, these enzymes and their metabolic products play crucial roles in immune regulation, tumor evasion, autoimmune diseases, and nervous system functions and have become targets for drug development [3,5,11].

Compared to the research on mammalian IDOs and TDO2, research on chicken IDO2 and TDO2 remains stagnant. Current research on chicken IDO2 and TDO2 is based on studies conducted in mammals. However, Trp decomposition in mammals mainly relies on IDO1 and TDO2; in contrast, chickens (and birds in general) lack the IDO1 gene, making IDO2 and TDO2 the only two dioxygenases that can initiate the kynurenine pathway [12]. The affinity of IDO2 for Trp and the efficiency of the in vitro enzymatic reaction are very low, differing greatly from those of IDO1 [13,14,15]. Researchers have found that the Trp content in chicken plasma is higher than that in humans (chicken Trp: 105.69 ± 9.20 μmol/L, human Trp: 67.4 ± 1.02 μmol/L), but the kynurenine content is lower than that in humans (chicken kynurenine: 0.34 ± 0.09 μmol/L, human kynurenine: 1.78 ± 0.04 μmol/L) [16,17]. Birds lack IDO1 (unique among vertebrates) and have higher plasma Trp but lower kynurenine levels than mammals [17], suggesting distinct Trp metabolism mechanisms. Because chickens lack IDO1, the IDO1-dependent metabolic and immune functions described in mammals must be mediated by IDO2 and/or TDO2 in birds. Nevertheless, the biochemical properties, tissue distribution, and physiological regulatory functions of avian IDO2 and TDO2 remain largely uncharacterized, creating critical gaps in avian physiology, immunology, and comparative research that warrant systematic investigation.

Chicken IDO2 and TDO2 may affect poultry production, welfare, and immunity. In nutritional metabolism, Trp is used for protein synthesis or broken down via the KP, determining its utilization efficiency. TDO2, the main catabolic enzyme, may affect the growth rate and feed conversion ratio of chickens based on its activity. In stress response and behavior, mammalian studies have shown that TDO2 is related to anxiety [18]. Poultry exhibit stress reactions under intensive farming, showing behaviors such as feather pecking and cannibalism [17]. We therefore hypothesize that, because Trp is a precursor of 5-hydroxytryptamine, chicken TDO2 activity may be associated with stress-related behaviors, which could provide targets for improving animal welfare; however, this hypothesis remains to be tested directly in birds. In immunomodulation, mammalian IDO1 is an inflammation-related gene that is upregulated during inflammatory responses [19]. Although chickens lack IDO1, the roles of chicken IDO2 and TDO2 in inflammation remain unknown.

Therefore, a systematic characterization of the basic biochemical properties and physiological regulatory functions of chicken IDO2 and TDO2 is of far-reaching significance for poultry and comparative immunological research. In the present study, we aimed to (i) characterize the tissue distribution, biochemical characteristics, and catalytic parameters of chicken IDO2 and TDO2; (ii) evaluate the effects of dietary Trp supplementation on hepatic IDO2/TDO2 expression and activity; and (iii) investigate the involvement of IDO2 and TDO2 in the inflammatory response. Specifically, we tested four hypotheses: (H1) dietary Trp supplementation increases hepatic IDO2/TDO2 catalytic activity; (H2) the Trp-induced increase in enzyme activity occurs without major changes in gene/protein abundance, indicating post-translational regulation; (H3) IDO2 and TDO2 are involved in the avian inflammatory response; and (H4) TDO2 has greater catalytic significance than IDO2 in avian Trp catabolism.

## 2. Materials and Methods

### 2.1. Animal Experiment

**Figure 1:**
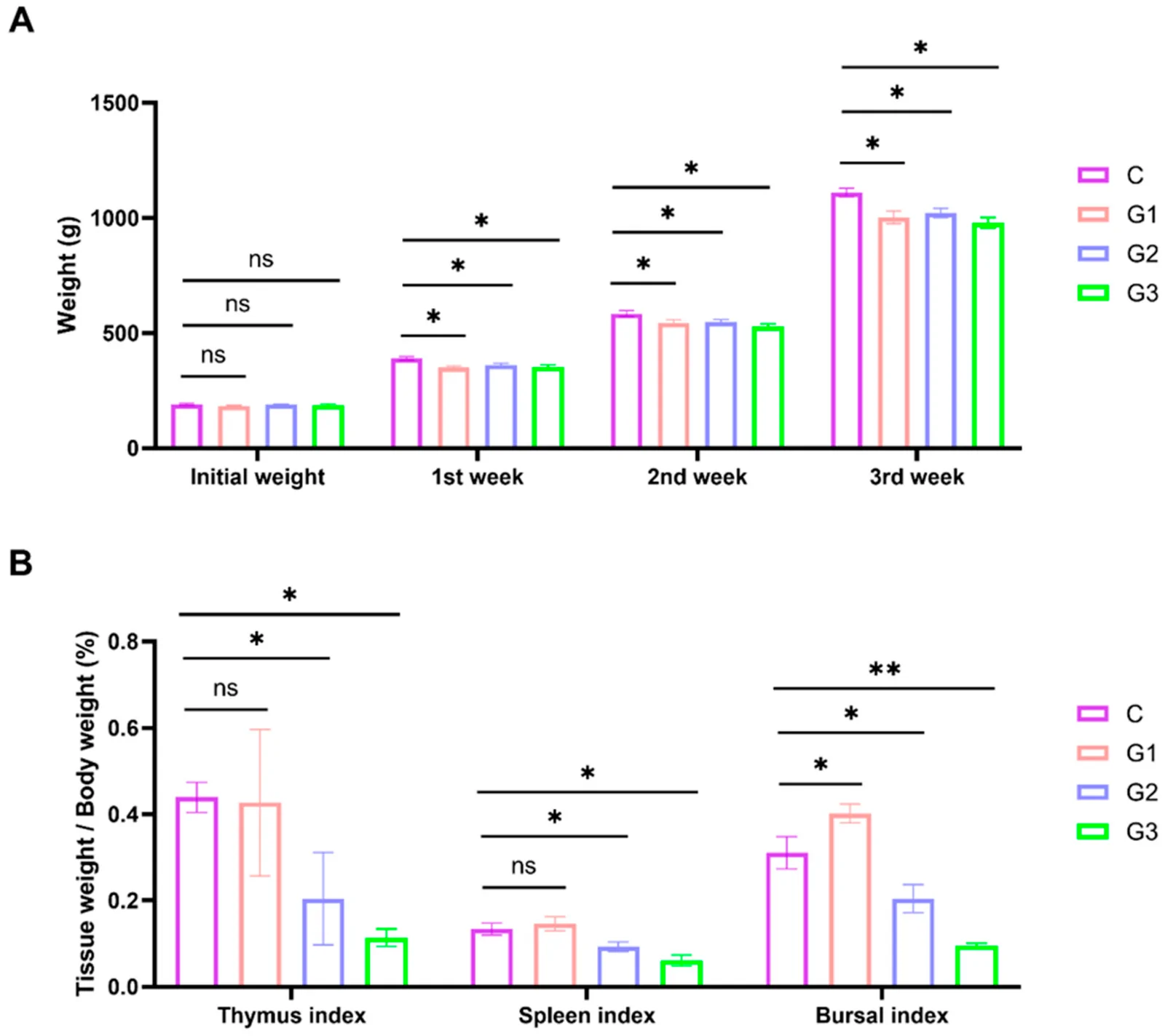
Effect of dietary L-Trp on body weight and immune organ index of chickens. (**A**) Chicken weekly body weight (*n* = 20 per group). (**B**) Immune organ index = organ weight× 100/body weight (*n* = 8 per group). The ‘C’ in the upper right corner represents the basal diet group, ‘G1’ represents basal diet + 0.1% Trp group, ‘G2’ represents basal diet + 0.2% Trp group, and ‘G3’ represents basal diet + 0.3% Trp group (the same applies below figure, no further comments). Data: mean ± SE. * *p* < 0.05, ** *p* < 0.01; ns, non-significant. Statistical comparisons were performed between groups at each time point.

One-day-old Partridge Shank chickens (*n* = 100) from Wugang Farm, Henan Province, China, were selected for a 10-day preliminary experiment, during which they were fitted with ankle rings, beaked, and fed a basal diet. The basal diet was formulated to contain 0.20% Trp, and the analysed Trp concentration was 0.23% (Table S1). The nutrient composition of the basal diet is shown in Table S1. At the end of the preliminary experiment, 20 chicks with poor growth, abnormal health status, or visible abnormalities were excluded, and the remaining 80 healthy chicks were randomly divided into four groups of 20 chicks each according to their body weight; exclusion was performed before randomization and was independent of any treatment-related characteristics. A formal experiment with Trp added to the diet was conducted for three weeks (21 days). The four groups in the formal trial were the basal diet group (group C) and groups fed the basal diet supplemented with an additional 0.1% (group G1), 0.2% (group G2), or 0.3% (group G3) L-Trp. L-Trp was added on top of the basal diet without replacing any other dietary component, yielding final formulated total Trp concentrations of approximately 0.30%, 0.40%, and 0.50% for groups G1, G2, and G3, respectively. Feed-grade L-Trp (purity >98%, cat no: 895689) was purchased from Hongrong Biotechnology Co., Ltd. (Zhengzhou, China). The chickens were fed ad libitum, and all 20 birds in each group were weighed weekly during the 3-week formal trial (Figure 1A). At the end of the formal experiment, eight chickens from each group were randomly selected and sacrificed, and their thymus, spleen, and bursa were weighed. The immune organ index (immune organ weight × 100/body weight) was calculated. At least 50 mg of duodenal contents and 300 μL of plasma were used for subsequent Trp-targeted metabolomic analysis and detection of glucose and triglycerides in the blood, and approximately 2 g of liver tissue was stored in liquid nitrogen for the detection of transcriptome, gene, and protein expression levels and IDO2 and TDO2 enzyme activity. The remaining liver samples were placed in 4% paraformaldehyde solution for subsequent wax sectioning and Oil Red O staining. An additional 15 organ samples (heart, liver, lung, spleen, bursa, thymus, pancreas, glandular stomach, brain, hypothalamus, colon, duodenum, ovary, pectoral muscle, and leg muscle) weighing approximately 1 g each were collected from one more chicken and preserved in liquid nitrogen for subsequent detection of the distribution of IDO2 and TDO2 in the chicken organs. As this tissue-distribution survey was based on a single individual, it was treated as an exploratory analysis rather than a population-level characterization.

### 2.2. Ethical Approval

All animal experimental protocols followed the “Instructive Notions with Respect to Caring for Laboratory Animals” issued by the Ministry of Science and Technology of China, and received ethical approval from the Animal Welfare and Research Ethics Committee of Henan Agricultural University (Approval Code: HNND2024031807; Approval Date: 18 March 2024). All animal operations complied with the ARRIVE 2.0 reporting guidelines.

### 2.3. Trp-Targeted Metabolism Detection

Trp and its related metabolites in chicken duodenal contents and plasma were detected using liquid chromatography–mass spectrometry (LC-MS/MS), as previously described [20]. After the sample was thawed, 50 mg (duodenal contents)/50 µL (plasma) of the sample was weighed and extracted with 500 µL/250 µL of methanol. An internal standard mixed solution (20 µL/10 µL; 250 ng/mL) was added to the extract for quantification. Subsequently, the extract was vortexed for 3 min, incubated in a refrigerator at −20 °C for 30 min, centrifuged at 12,000× *g* and 4 °C for 10 min, and 250 µL/150 µL of the supernatant was collected. The supernatant was re-centrifuged at 12,000× *g* and 4 °C for 5 min. The supernatant (150 µL/100 µL) was analyzed using ultra-high-performance liquid chromatography-tandem mass spectrometry (UHPLC-MS/MS) (UPLC, ExionLC AD; MS, QTRAP 6500+ System). Chromatographic separation was performed on a column (Waters ACQUITY UPLC HSST3, 100 mm × 2.10 mm, 1.8 µm, Waters, Milford, MA, USA) maintained at 40 °C. The analytes were eluted using a gradient of 0.1% formic acid in water (solvent A) and 0.1% formic acid in acetonitrile (solvent B). The flow rate was 0.35 mL/min, and the injection volume was 5 µL. The mass spectrometry detection conditions were as follows: the electrospray ionization source temperature was 550 °C, the ion spray voltages (IS) were 5500 V (positive) and −4500 V (negative), and the curtain gas (CUR) was set at 35 psi. Trp and its related metabolites were analyzed using scheduled multiple reaction monitoring (MRM). Data acquisition was performed using Analyst 1.6.3 software (Sciex). MultiQuant 3.0.3 software (Sciex) was used to quantify all metabolites.

### 2.4. Polymerase Chain Reaction (PCR)

As the distribution of IDO2 and TDO2 in various chicken organs was unclear, a distribution analysis of IDO2 and TDO2 was first performed in this study. The PCR primer sequences for IDO2, TDO2, and GAPDH are listed in Supplementary Table S2. RNA was extracted from 15 chicken tissues (heart, liver, lung, spleen, bursa, thymus, pancreas, glandular stomach, brain, hypothalamus, colon, duodenum, ovaries, pectoral muscles, and leg muscles), followed by reverse transcription to obtain cDNA, which was then amplified by PCR to determine whether the gene of interest was expressed in the electrophoresis band. At the same time, the GAPDH gene was amplified as a positive control. PCR was performed according to the manufacturer’s instructions provided with the kit (Accurate Cat. AG12301, Changsha, China). The expression of IDO2 and TDO2 in various chicken organs was analyzed, and the liver was selected for further analysis.

### 2.5. Transcriptome Sequencing

Total RNA was extracted from each liver tissue sample using TRIzol reagent (Invitrogen, Carlsbad, CA, USA) according to the manufacturer’s instructions. Equal amounts of RNA from two individual chickens in the same group were pooled to form one RNA library using the TruSeq Stranded Total RNA Library Prep Kit (Illumina, San Diego, CA, USA) according to the manufacturer’s instructions; thus, each of the four libraries per dietary group represented two pooled chickens (i.e., eight individual chickens per group contributed to four pooled libraries). Each pooled library was treated as one experimental unit for subsequent statistical analysis (*n* = 4 libraries per group). Then, 16 small RNA libraries (C_1, C_2, C_3, C_4, G1_1, G1_2, G1_3, G1_4, G2_1, G2_2, G2_3, G2_4, G3_1, G3_2, G3_3, and G3_4) were prepared. Double-end sequencing was performed using an Illumina HiSeq Xten (Illumina, San Diego, CA, USA) according to the manufacturer’s instructions. Sequencing adapters, low-quality reads, fragments <50 bp in length, and bases with an average quality value of <20 (Q20) were filtered out using fastp software. Using Bowtie2 software, the filtered data were aligned to the rRNA database to remove rRNA sequences and obtain clean data for further analysis. The clean data were aligned to the reference genome (Gallus bGalGal1.mat.broiler. GRCg7b) using hisat2. The FPKM values were calculated and used to compare the differences in gene expression between the different samples. Differentially expressed genes (DEGs) were initially screened using |Fold Change| > 1.4 and *p* < 0.05. Because the dietary intervention induced modest but consistent transcriptomic changes, the |Fold Change| > 1.4 threshold was adopted to capture biologically relevant changes in metabolic and immune pathways; to control the false discovery rate arising from the large number of tested genes, the identified DEGs were further filtered using an FDR-adjusted *p*-value (adjusted *p* < 0.05, Benjamini–Hochberg method). The Pearson correlation coefficient was calculated to analyze the correlations between biological replicates and visualized using a heatmap. GO and KEGG analyses of the DEG were performed using the R package clusterProfiler (http://bioconductor.org/packages/release/bioc/html/clusterProfiler.html (accessed on 21 June 2024).

In addition, following the overexpression of chicken IDO2 and TDO2 in LMH cells (Leghorn Male Hepatoma, the chicken hepatocellular carcinoma cell line), the extracted RNA was used to construct 18 small RNA libraries. They included 3 biological replicates per group: overexpression negative control (oeNC-1, oeNC-2, oeNC-3), IDO2 overexpression (oe_IDO2-1, oe_IDO2-2, oe_IDO2-3), TDO2 overexpression (oe_TDO2-1, oe_TDO2-2, oe_TDO2-3), silencing negative control (siNC-1, siNC-2, siNC-3), IDO2 silencing (si_IDO2-1, si_IDO2-2, si_IDO2-3), and TDO2 silencing (si_TDO2-1, si_TDO2-2, si_TDO2-3). DEGs were initially screened using |Fold Change| > 1.4 and *p* < 0.05 and were further filtered using an FDR-adjusted *p*-value (adjusted *p* < 0.05, Benjamini–Hochberg method) to control for multiple testing. Following the above method, GO and KEGG enrichment analyses were performed on the DEG.

### 2.6. Quantitative Real-Time PCR

Liver RNA was extracted and reverse transcribed as described above. The qPCR primers used are listed in Supplementary Table S3. qPCR was performed using the SuperReal PreMix Plus (Tiangen, Beijing, China) according to the manufacturer’s instructions. Gene expression was normalized to that of ACTB. The relative expression of each gene was calculated using the 2−ΔΔCt method. The relative expression level of each gene was measured in triplicate, and the average value was used for statistical analysis.

### 2.7. Western Blot

Total protein was extracted from the liver using RIPA buffer (Sangon Biotech, Shanghai, China). After protein quantification, equal amounts of protein samples were loaded and subjected to 10% SDS-PAGE for separation, followed by transfer onto 0.45 µm NC membranes (Solarbio, Beijing, China). After blocking with Protein-Free Rapid Blocking Buffer (Epizyme, Shanghai, China) for 10 min, the membranes were incubated overnight at 4 °C with primary antibodies. Secondary antibodies were applied for incubation at room temperature (approximately 25 °C for 2 h) after washing twice with TBST. Protein bands were visualized using a highly sensitive enhanced chemiluminescence (ECL) reagent (Sangon Biotech, Shanghai, China) and analyzed using ImageJ software. The antibodies used in this study are listed in Supplementary Table S4.

### 2.8. Total Enzyme Activity Assay of IDO2 and TDO2 Ex Vivo

The liver samples were processed as follows. The liver tissue was weighed using an electronic balance, and phosphate-buffered saline (PBS) solution was added in proportion, with a tissue weight to PBS ratio of 1 g: 9 mL. The tissue was ground on ice thoroughly, centrifuged for 20 min at 2000× *g* and 4 °C, and the supernatant was collected. Notably, this tissue homogenate assay detects combined total dioxygenase activity of hepatic IDO2 and TDO2 and cannot individually quantify the catalytic capacity of each isoform; separate purified recombinant protein kinetic assays (Section 2.13) were conducted to distinguish intrinsic enzymatic properties of the two proteins. The reaction mixture was prepared as previously described [12,21,22]. The enzyme reaction mixture was 200 μL and contained 50 μL of tissue supernatant, 50 mM phosphate buffer, 5 mM ascorbic acid, 100 μg/L catalase, 10 μM methylene blue, and 250 μM L-Trp substrate. The reaction mixture was incubated at 37 °C for 60 min and was terminated by adding 40 μL of trichloroacetic acid (30% *w*/*v*). Subsequently, the samples were incubated at 50 °C for 30 min to hydrolyze N-formylkynurenine to kynurenine. After centrifugation for 5 min at 10,000× *g*, the supernatant was transferred to a new microplate and mixed with an equal volume of p-dimethylaminobenzaldehyde in acetic acid (2% *w*/*v*). The absorbance of the yellow complexes derived from kynurenine was measured at 480 nm using a spectrophotometer. One unit of enzyme activity (U) was defined as the generation of 1 nmol/h of kynurenine [23].

### 2.9. Oil Red O Staining

Oil Red O staining of the liver was performed as described below. Fresh liver tissue was frozen in liquid nitrogen for 15 s and then placed in a −80 °C refrigerator. The sample was then removed, embedded in an OCT embedding agent (Servicebio, Wuhan, China), and sectioned at 8–10 μm thickness. After 8–10 min of staining with freshly prepared saturated Oil Red O solution (Servicebio, Wuhan, China), the cells were re-stained with hematoxylin for 3–5 min, the slides were sealed with glycerin gel, and observed under a microscope. Images were collected using the ImageJ software 1.53a vesrion.

### 2.10. Plasma Glucose and Triglyceride Assays

Glucose in chicken plasma was detected using a Glucose Kit (Mindray Biotech, Shenzhen, China). The assay was performed using a biochemistry analyzer (Mindray BS Series, Shenzhen, China) and the glucose oxidase method, according to the manufacturer’s instructions. Triglycerides in chicken plasma were detected using a Triglyceride Kit (Mindray Biotech, Shenzhen, China) using the Glycerol-3-phosphate dehydrogenase (GPD)-peroxidase (POD) method, according to the manufacturer’s protocols.

### 2.11. Molecular Docking

Molecular docking of protein-ligand interactions was performed using AlphaFold 3 (https://alphafoldserver.com) and SwissDock (https://www.swissdock.ch/). The amino acid sequences of chicken IDO2 (XM_040689374.2) and TDO2 (ENSGALT0000154649.1) were retrieved from the NCBI and Ensembl databases. AlphaFold 3 was used to predict the three-dimensional structures of chicken IDO2 and the chicken TDO2 monomer and to perform protein–ligand docking of the cofactor heme b and the substrate L-Trp with the predicted structures in a Python 3.11 environment by executing the run_alphafold.py script; ligand protonation states and partial charges were assigned using the default AlphaFold 3/SwissParam protocols, and heme b was included as a cofactor during docking. The docking of L-Trp and heme b was cross-validated using SwissDock, from which the attracting cavity (AC) and SwissParam scores were obtained; more negative AC and SwissParam scores indicate more favorable predicted binding. Owing to the high complexity of the TDO2 tetramer, docking scores could be calculated only for the TDO2 monomer. The resulting models were visualized using UCSF Chimera software 1.2.0.

### 2.12. Plasmid Construction

Total RNAs (from Partridge Shank chicken liver and the human HepG2 cell line) was extracted, and cDNAs synthesis was performed as described above. Based on chicken IDO2 (XM_040689374.2), chicken TDO2 (ENSGALT0000154649.1), and human IDO1 (NM_005651.4) CDS sequences in the NCBI and Ensembl databases, the primers used are listed in Supplementary Table S5. Human IDO2 (ENST0000052986.4) and TDO2 (ENST00000536354.3) CDS were synthesized directly by Sangon Biotech Co., Ltd. (Shanghai, China). Human IDO2/TDO2 CDS sequences were verified via sequencing with 100% identity to NCBI references (ENST0000052986.4/ENST00000536354.3). Following the instructions of the ClonExpress II One-Step Cloning Kit (Vazyme, Nanjing, China), the amplified products were cloned into a pGEX-4T-3 vector. The nucleotide sequences of the constructs were confirmed by sequencing. These plasmids were used for protein expression and purification of IDOs and TDO2.

The same method described above was used to construct two overexpression plasmids by inserting the CDS sequences of chicken IDO2 (XM_040689374.2) and TDO2 (ENSGALT0000154649.1) into a suitable enzyme cutting site for the pcDNA3.1-3×FLAG vector (primers in Table S5).

### 2.13. Expression and Purification of Recombinant Proteins

GST-fusion proteins were expressed in *E. coli* BL21(DE3) cells. The transformed bacteria were grown in Luria–Bertani (LB) liquid medium with ampicillin overnight at 37 °C until the absorbance at 600 nm reached approximately 0.6. Expression was induced with 0.5 mM IPTG (Isopropyl β-D-1-thiogalactopyranoside) and 7 μM hemin for 8 h at 28 °C. The pellet from 250 mL of the bacterial culture was harvested by centrifugation, suspended in 30 mL of PBS (pH 7.4), and sonicated. The sample was centrifuged at 12,000× *g* for 15 min at 4 °C. The clear supernatant was filtered through a 0.22 μm filter and directly applied to a GSTrap FF column (1 mL, Cytiva, Shanghai, China). After washing with 10 mL PBS, GST-fusion IDOs or TDO2 were eluted with 50 mM Tris-HCl (pH 8.0) and 10 mM reduced glutathione. Proteins eluted with GST-tags were used for enzyme activity assays.

In addition, the GST tag of the fusion protein was also removed. After the supernatant of cell lysis was applied to a GSTrap FF column, GST-fusion IDOs or TDO2 were cleaved using thrombin protease (1 cleavage units/100 μg fusion protein, MedChemExpress, Shanghai, China) at 4 °C for 16 h in PBS buffer by on-column cleavage. After thrombin digestion, the cleaved mixture was applied to a benzylpenicillin agarose gel gravity column (Solarbio, Beijing, China) to remove the thrombin. Purified proteins were used for the enzyme activity assays.

### 2.14. Enzyme Parameters Assays

Recombinant IDOs and TDO2 activities were analyzed based on the total enzyme activity ex vivo, as described above. The enzyme reaction mixture was the same, except that the substrates (L-Trp, D-Trp, 5-OH-Trp, and tryptamine) were prepared with the maximum amount of substrate that could be dissolved in the mixture. Take 150 μL of enzyme reaction mixture solution containing the substrate, then add 50 μL of enzyme solution (purified protein) to make the final volume up to 200 μL for enzyme activity determination. The termination of the enzymatic reaction and pigment determination were the same as those in the total enzyme activity assay. The temperature of the enzyme reaction was chosen as 37 °C and 42 °C, which are adapted to the body temperature of mammals and birds, respectively. The solution pH was adjusted by changing the ratio of potassium dihydrogen phosphate to potassium hydrogen diphosphate.

Simultaneously, the inhibition constants (Ki) of chicken IDO2 and TDO2 were measured. Referring to previous studies [12,24], 1-methyl-L-tryptophan (L-1MT) and 1-methyl-D-tryptophan (D-1MT) were used as IDO2 inhibitors to determine their inhibitory effects on the chicken IDO2 protein, and the inhibitory effect of a commercially available human TDO2 inhibitor, 680C91, on the chicken TDO2 protein was measured. The structures of the three inhibitors are shown in Supplementary Figure S1. The 200 μL reaction mixture comprised the following components: 50 mM phosphate buffer, 5 mM ascorbic acid, 100 μg/L catalase, 10 μM methylene blue, different concentrations of L-Trp substrate, different concentrations of inhibitor solution, and 50 μL purified protein. Four concentrations of the IDO2 inhibitor were used: 126, 63, 31.5, and 0 μM. Four concentrations of TDO2 inhibitors were used: 1000, 500, 250, and 0 nM.

### 2.15. Cell Culture and Transfection

LMH cells were cultured in complete LMH cell medium (Beyotime, Shanghai, China) at 37 °C with 5% CO2. Transient transfection with the overexpression plasmid and small interfering RNA (Table S6) was performed using Lipofectamine 3000 reagent (Thermo Fisher Scientific, Shanghai, China), following the manufacturer’s instructions for the overexpression and knockdown of chicken IDO2 and TDO2. The construction of the overexpression plasmid for chicken IDO2/TDO2 was described in the previous section on plasmid construction. For IDO2 knockdown experiments, only one small interfering RNA sequence was designed and only this one was transfected. For TDO2 knockdown experiments, three small interfering RNA sequences were designed and transfected together. The treatment groups were transfected with overexpression plasmids or small interfering RNAs according to the experimental objectives. In the overexpression experiments of chicken IDO2/TDO2, pcDNA3.1-3×FLAG empty vector was used as the control (oeNC group), while si-NC (Table S6) was used as the control (siNC group) in the knockdown experiments. Transfection experiments included 3 biological replicates and 3 technical replicates. For statistical analysis, we first calculated the mean value of the 3 technical replicates for each biological replicate, resulting in 3 independent data points (one mean value per biological replicate, *n* = 3). After treatment for 24 and 36 h, mRNA and protein expression levels were assessed using RT-PCR and Western blotting, respectively. Transcriptome sequencing was performed on cells transfected for 24 h, as described above.

### 2.16. Establishment of an Lipopolysaccharide (LPS) -Induced Inflammatory Model in LMH Cells

LMH cells were cultured in vitro and treated with different concentrations of LPS (10, 40, 80, and 120 μg/mL) for 24 h to establish an LPS-induced inflammatory model. Each LPS concentration was tested in 8 biological replicates with 3 technical replicates. The expression levels of inflammatory cytokines (IL-1β and TNF-α) in the cells were detected using RT-PCR (primers listed in Table S3). The experimental group, in which inflammatory factors were significantly upregulated compared to the control group, was used to determine whether the total enzymatic activities of chicken IDO2 and TDO2 underwent significant changes. The method for detecting total enzyme activity was as described in the above-mentioned section of the total enzyme activity assay for IDO2 and TDO2 ex vivo. Of note, an independent cell-viability/cytotoxicity assay (e.g., CCK-8, MTT, or LDH-release assay) was not performed at the tested LPS concentrations; this limitation is further discussed in Section 4.

### 2.17. Chicks’ Inflammation Caused by Avian Pathogenic Escherichia coli (APEC)

In this trial, 60 one-day-old chicks were divided into two groups: control and treated (infected with APEC). The chicken challenge method with APEC was based on a previous study [25]. Briefly, each group of 30 chicks was housed in a negative-pressure isolation cabin for an adaptation period of eight days. The formal trial began on the 9th day. APEC strains (O1, O2, O78) were cultured in LB medium to log phase (OD600 = 0.6), and concentrations were verified via colony counting. The chicks in the treatment group were intramuscularly injected with 0.5 mL of an equal mixture of APEC O1, O2, and O78 at a concentration of 1 × 10^8^ CFU/mL [25]. Infected chicks were monitored for clinical signs (lethargy, feed intake, diarrhea) at 24, 48, and 72 h post-infection. Twelve birds per group were randomly selected and sacrificed 3 days after the APEC challenge. Then, the pathological anatomy was analyzed to confirm systemic inflammation, and blood and liver samples were collected. Blood samples were used to detect inflammatory factors (IL-1β and TNF-α) according to the ELISA Kit (Cusabio, Wuhan, China) Instruction Manual. Liver samples were used to determine the total activities of chicken IDO2 and TDO2.

### 2.18. Statistical Analysis

Data are presented as mean ± SEMs. A unpaired *t*-test in GraphPad Prism 9.0 was used for pairwise comparison analysis (C vs. G1, C vs. G2, C vs. G3). Multiple group comparisons were performed using ANOVA with GraphPad Prism 9.0. All statistical tests (e.g., *t*-test, ANOVA) were performed using the biological replicate means. Statistical significance was set at *p* < 0.05. Weekly body-weight data were analyzed by between-group comparisons at each individual time point (unpaired *t*-test/ANOVA); within-bird correlation across weeks was not modeled, because the study hypotheses concerned between-group differences rather than within-individual longitudinal trajectories. Metabolites in Trp-targeted metabolism with variable importance in the projection (VIP) values >1 and *p* < 0.05 were selected as the differential metabolites. VIP values were extracted from the orthogonal partial least squares discriminant analysis (OPLS-DA) results, which also contained score plots and permutation plots, and were generated using the R package MetoboAnalystR. DEGs from the liver transcriptome were screened using |Fold Change| > 1.4 and *p* < 0.05 and were further filtered using an FDR-adjusted *p*-value (adjusted *p* < 0.05, Benjamini–Hochberg method) to control for multiple testing. Enzyme kinetic parameters (Km, Vmax, Kcat, Ki) were calculated from three independent recombinant protein purification batches (*n* = 3 biological replicates) using the built-in Michaelis-Menten and competitive inhibition modules of GraphPad Prism 9.0.

## 3. Results

### 3.1. Effects of Dietary L-Trp on Chicken Body Weight and Immune Organ Index

Compared to the basal diet group (Group C), body weight gain of chickens in the three groups (G1, G2, and G3) supplemented with Trp showed a significant reduction in the first week (*p* < 0.05) and continued to show a significant decrease in the second and third weeks (*n* = 20 per group; Figure 1A). After three weeks, the thymus and spleen indices decreased with an increase in Trp addition, and groups G2 and G3 differed significantly from group C (*p* < 0.05). The bursa index of group G1 was significantly higher than that of group C, whereas those of groups G2 and G3 were significantly lower than that of group C (*n* = 8 per group; Figure 1B).

### 3.2. The Content of Trp-Related Metabolites in Chicken Duodenum and Plasma

**Figure 2:**
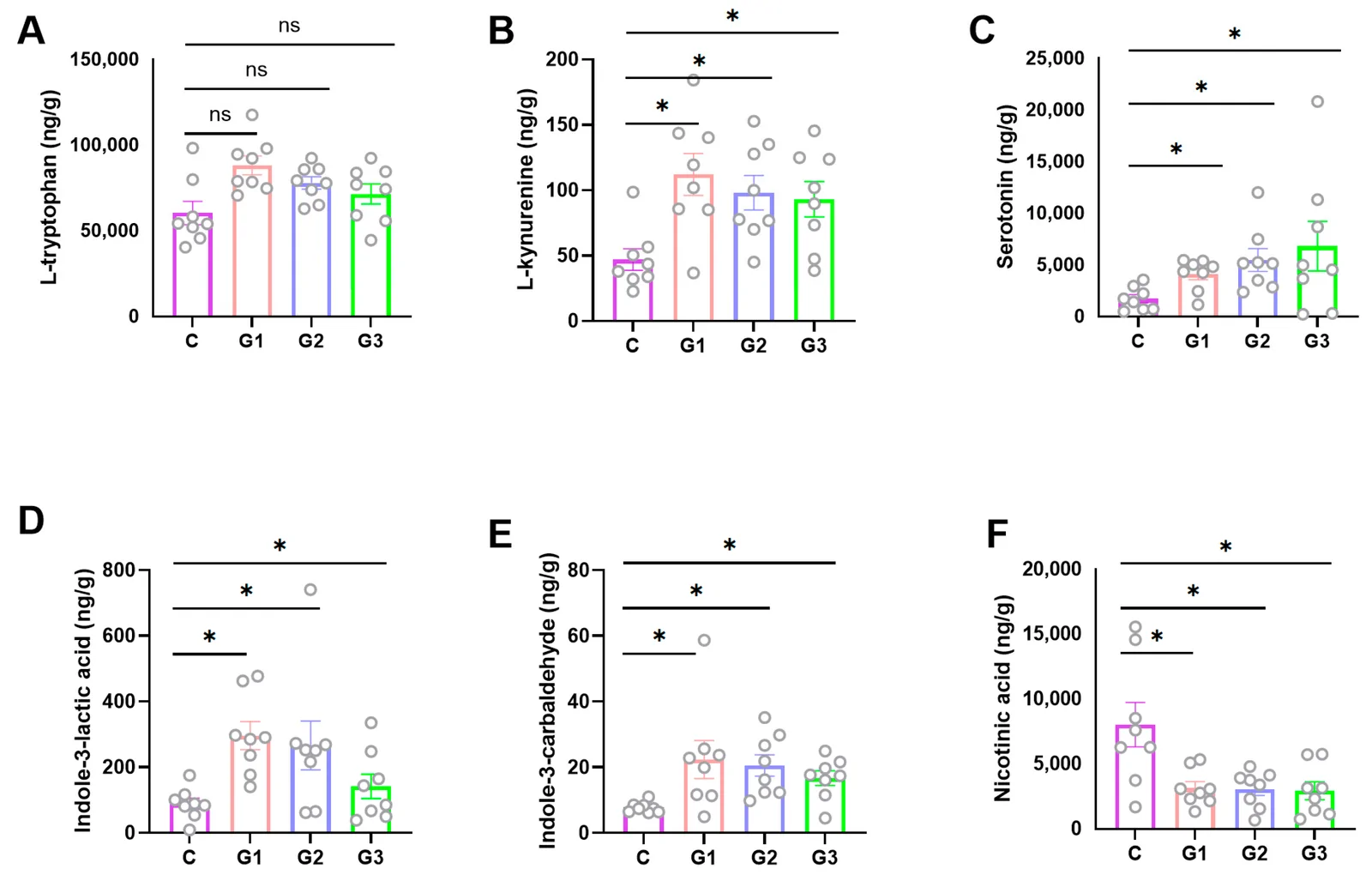
Duodenal differential Trp metabolites. (**A**) L-Trp. (**B**) L-kynurenine. (**C**) Serotonin. (**D**) Indole-3-lactic acid. (**E**) Indole-3-carbaldehyde. (**F**) Nicotinic acid. Markers with * satisfy VIP > 1, fold change >2 or <0.5; ns = no significance. All data are presented as mean ± SE (*n* = 8 per group).

A total of 31 Trp-related metabolites were quantified via LC-MS/MS. Duodenal L-Trp concentrations showed no inter-group differences (*p* > 0.05, Figure 2A). However, downstream kynurenine metabolites (L-kynurenine, serotonin, indole-3-lactic acid, and indole-3-carbaldehyde) were significantly accumulated in Trp supplementation groups (G1, G2, and G3 group) rather than Trp itself (C group) (*p* < 0.05, Figure 2B–E), except for nicotinic acid (*p* < 0.05, Figure 2F).

**Figure 3:**
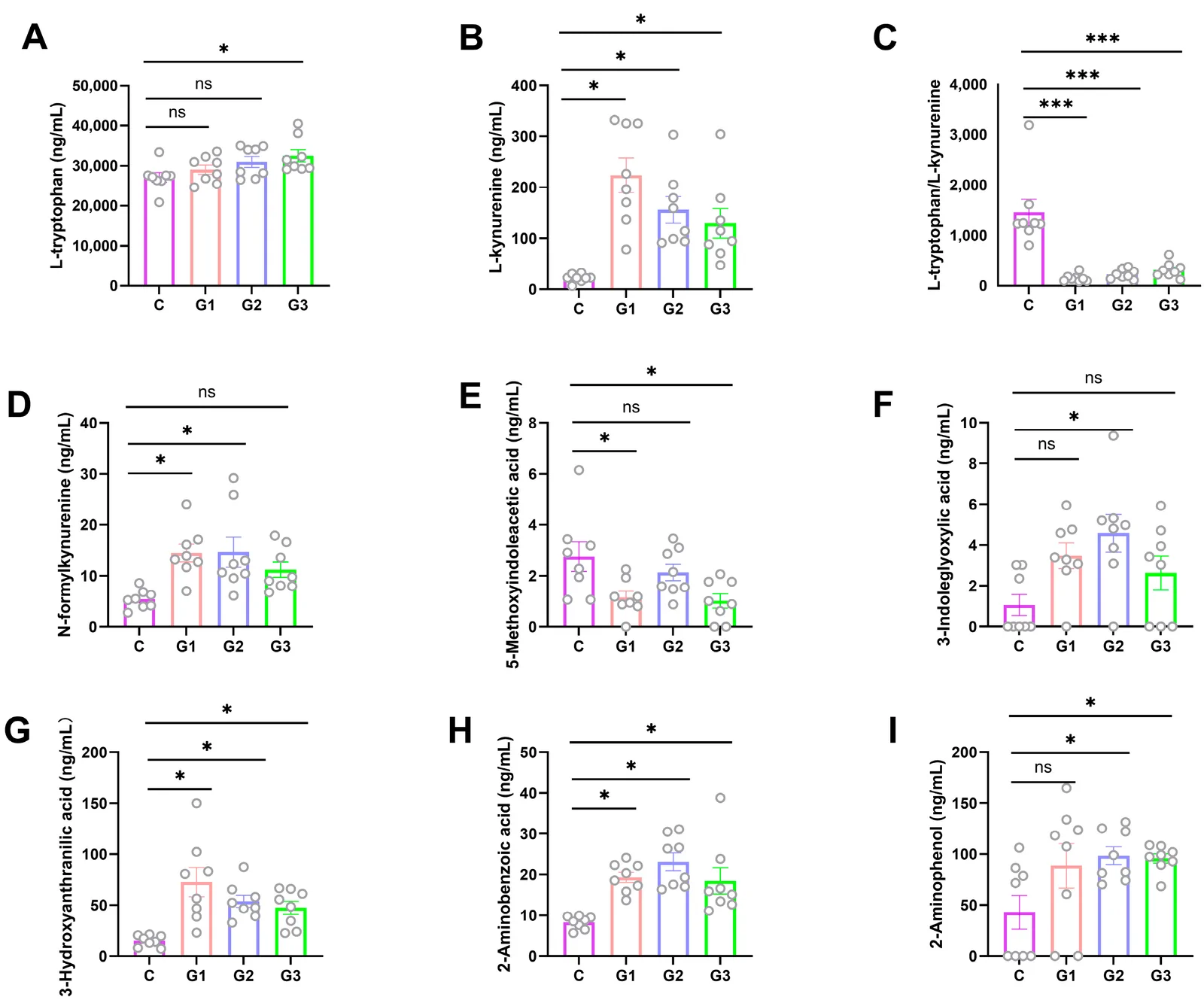
Differential metabolites in chicken plasma. (**A**) L-Trp. (**B**) L-kynurenine. (**C**) L-Trp/L-kynurenine. (**D**) N-formylkynurenine. (**E**) 5-Methoxyindoleacetic acid. (**F**) 3-Indoleglyoxylic acid. (**G**) 3-Hydroxyanthranilic acid. (**H**) 2-Aminobenzoic acid. (**I**) 2-Aminophenol. The metabolites in Trp-targeted metabolism with variable importance in the projection (VIP) values > 1 and fold changes > 2 or <0.5 were selected as differential metabolites, indicated by *. ns, no significant difference. Data from L-Trp/L-kynurenine were analyzed using one-way analysis of variance (ANOVA), and *p* < 0.001 is indicated by ***. All data are presented as mean ± SE (*n* = 8 per group).

In plasma, only the highest-dose G3 group exhibited elevated L-Trp (Figure 3A), whereas L-kynurenine was significantly increased across all supplemented treatments, accompanied by a reduced Trp/kynurenine ratio (Figure 3B,C). Most kynurenine pathway intermediates were upregulated without clear linear dose dependence (Figure 3D–I).

### 3.3. Effect of Dietary L-Trp on IDO2 and TDO2 Related Genes, Protein Expression, Total Enzyme Activity and Other Actions

In this exploratory analysis based on tissues from a single individual, IDO2 was detected in all 15 tested chicken tissues, whereas TDO2 was only detectable in the liver and thymus (Figure S2). Liver tissue was selected for multi-omics and functional detection.

**Figure 4:**
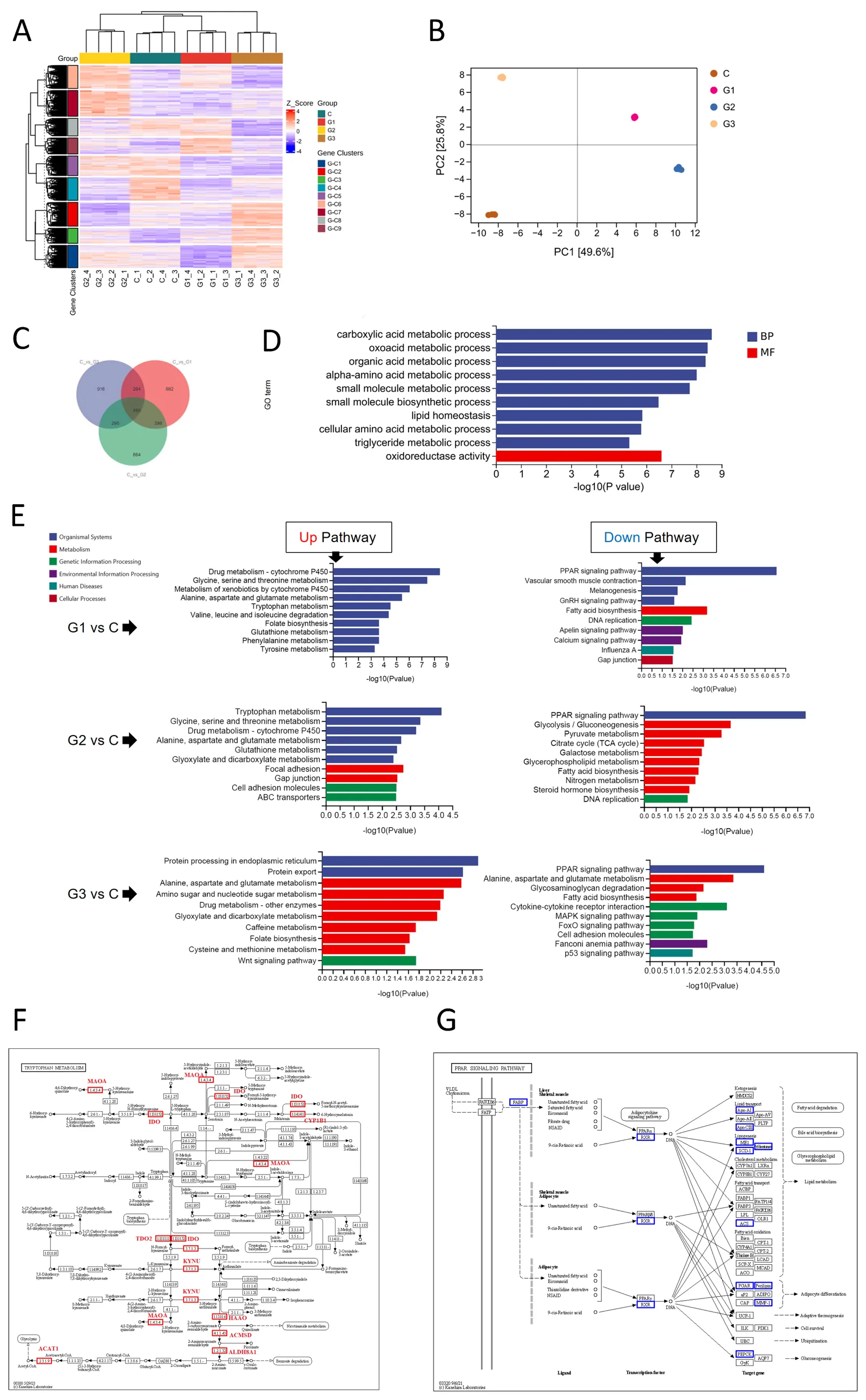
Effect of L-Trp on chicken liver transcriptome. (**A**) Cluster heat map of biological replicates for each group. (**B**) Principal component analysis (PCA) diagram of the biological replicates for each group. (**C**) Venn diagram of DGEs. (**D**) GO enrichment bar plot of common DGEs. (**E**) KEGG enrichment bar plots of the DGEs. (**F**) Trp metabolism in the KEGG database. The red dots indicate upregulated genes. (**G**) PPAR signaling pathway in the KEGG database. Blue indicates downregulated genes.

Transcriptome PCA and heatmap confirmed good intra-group replicate clustering (Figure 4A,B). A total of 682, 864, 916 DEGs were identified in G1/G2/G3 vs. C, with 465 shared differential genes (Figure 4C). GO enrichment centered on amino acid and lipid metabolic processes; KEGG analysis revealed upregulated kynurenine metabolism and suppressed PPAR/fatty acid biosynthesis pathways (Figure 4D–G). IDO2, TDO2 and KYNU were transcriptionally upregulated, while PPAR cascade lipid regulatory genes were downregulated. qPCR validation matched transcriptome trends (Figure S3).

**Figure 5:**
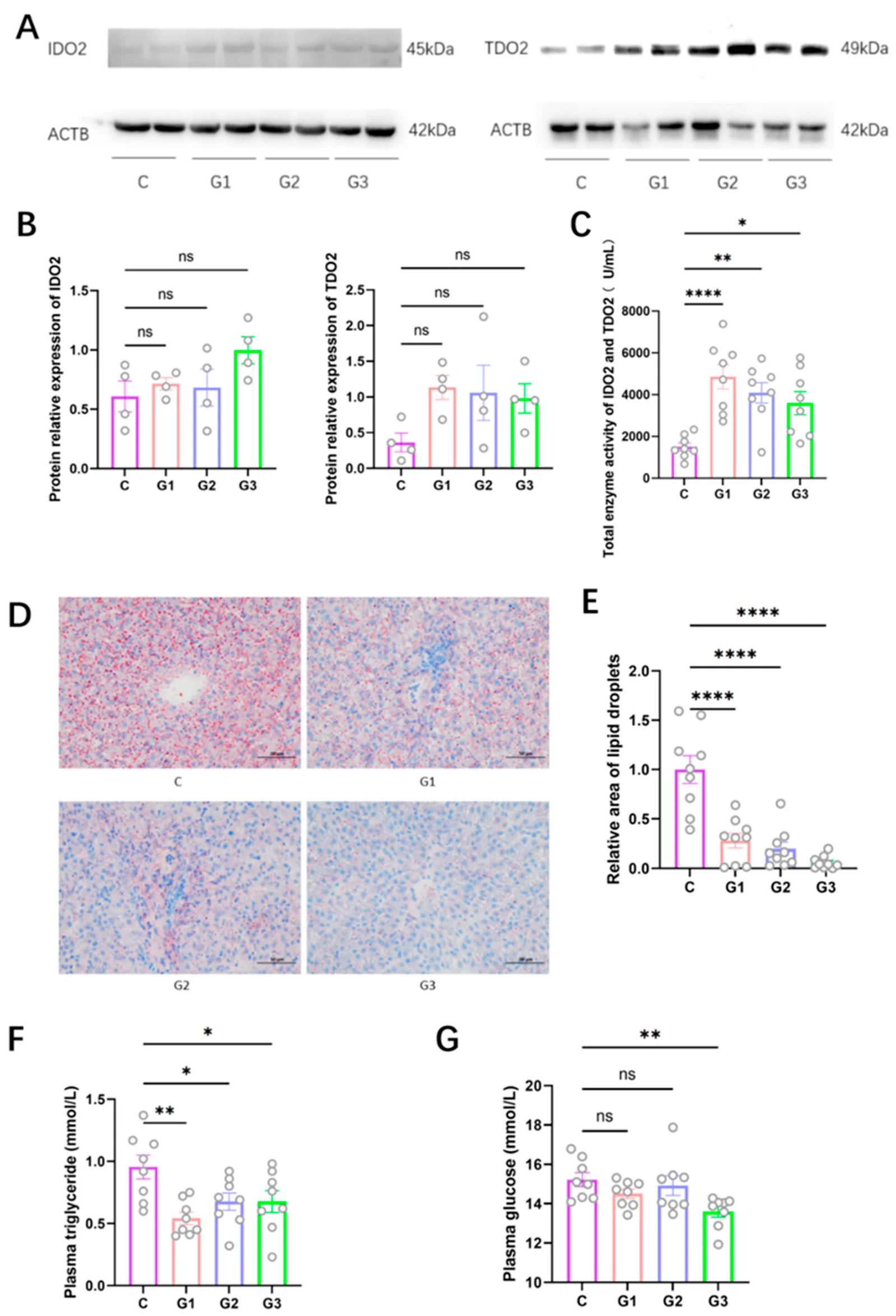
Effect of L-Trp on protein expression, total enzyme activity, fat content, and plasma indicators. (**A**) Western blot analysis of IDO2 and TDO2 proteins in chicken liver. (**B**) Protein expression levels of IDO2 and TDO2 (*n* = 4 per group). (**C**) Total enzyme activities of IDO2 and TDO2 in the chicken livers (*n* = 8 per group). (**D**) Oil Red O staining of each group (scale bars represent 50 µm). (**E**) Relative area of the lipid droplets. (**F**,**G**) Plasma triglyceride and glucose levels (*n* = 8 per group). * *p* < 0.05, ** *p* < 0.01, and **** *p* < 0.0001; ns, no significant difference.

No significant changes were observed in hepatic IDO2 and TDO2 protein abundance across treatments, while total combined catalytic activity increased dose-dependently with dietary Trp (Figure 5A–C). Consistent with PPAR pathway suppression, hepatic neutral lipid droplet area and circulating triglyceride concentrations decreased with rising Trp levels; only G3 exhibited reduced plasma glucose (Figure 5D–G).

### 3.4. Molecular Docking Model of Chicken IDO2 and TDO2 with L-Trp

**Figure 6:**
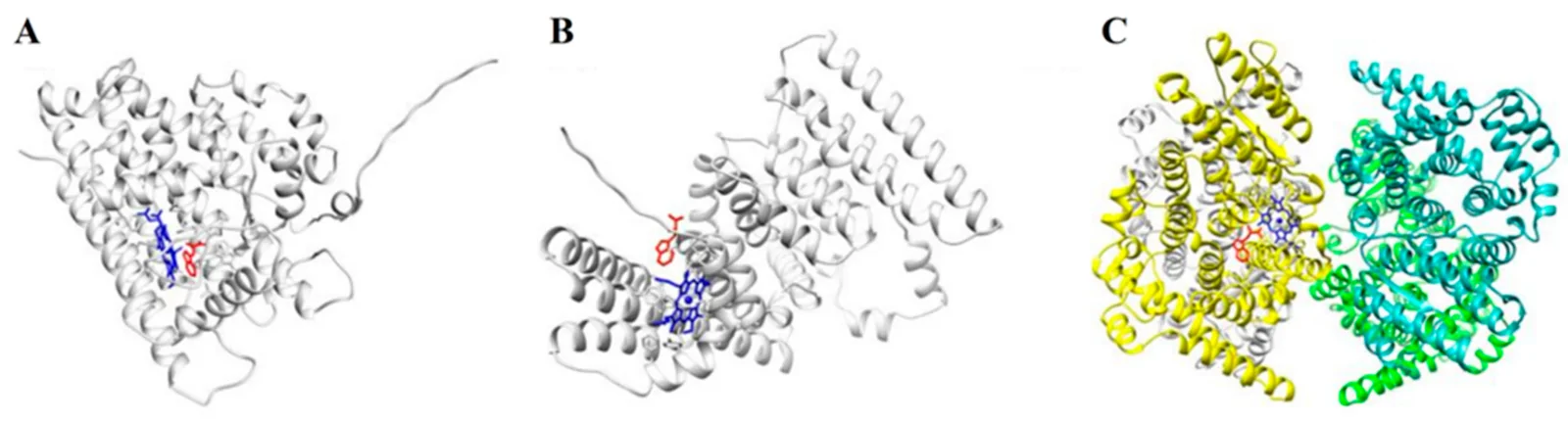
Molecular docking model of chicken IDO2 and TDO2 proteins with L-Trp. (**A**) IDO2 model. (**B**) TDO2 monomer model. (**C**) TDO2 tetramer model. White, yellow, green, and blue represent monomers. Blue represents heme b. The color red represents L-Trp.

The results of the molecular docking model are presented in Figure 6. The attracting cavity (AC) and SwissParam scores of IDO2 were 12.119671 and −7.1337, respectively (Figure 6A). The TDO2 monomers were 12.066832 and −7.2107, respectively (Figure 6B). Due to the high complexity of the TDO2 tetramer, the Swiss website failed to calculate its scores (Figure 6C).

### 3.5. Biochemical Analysis of Chicken IDO2 and TDO2

The recombinant vector with the correct sequence was used to express recombinant proteins in *E. coli* BL21(DE3). The recombinant protein with a GST tag was verified by Western blotting (Figure S4) and purified by affinity chromatography using a GSTrap FF column, and the purification results are shown in Figure S5A–E. Due to the large molecular weight of the GST tag (26 kDa), to more accurately study the enzymatic properties of the target protein, the GST tag was removed by on-column cleavage, thrombin was removed using benzylpenicillin agarose gel gravity column chromatography, and the protein purification results are shown in Figure S5F–J. Purified protein without a GST tag was used for subsequent enzyme activity analysis. The concentration of the purified protein was 0.1–1 mg/mL.

**Figure 7:**
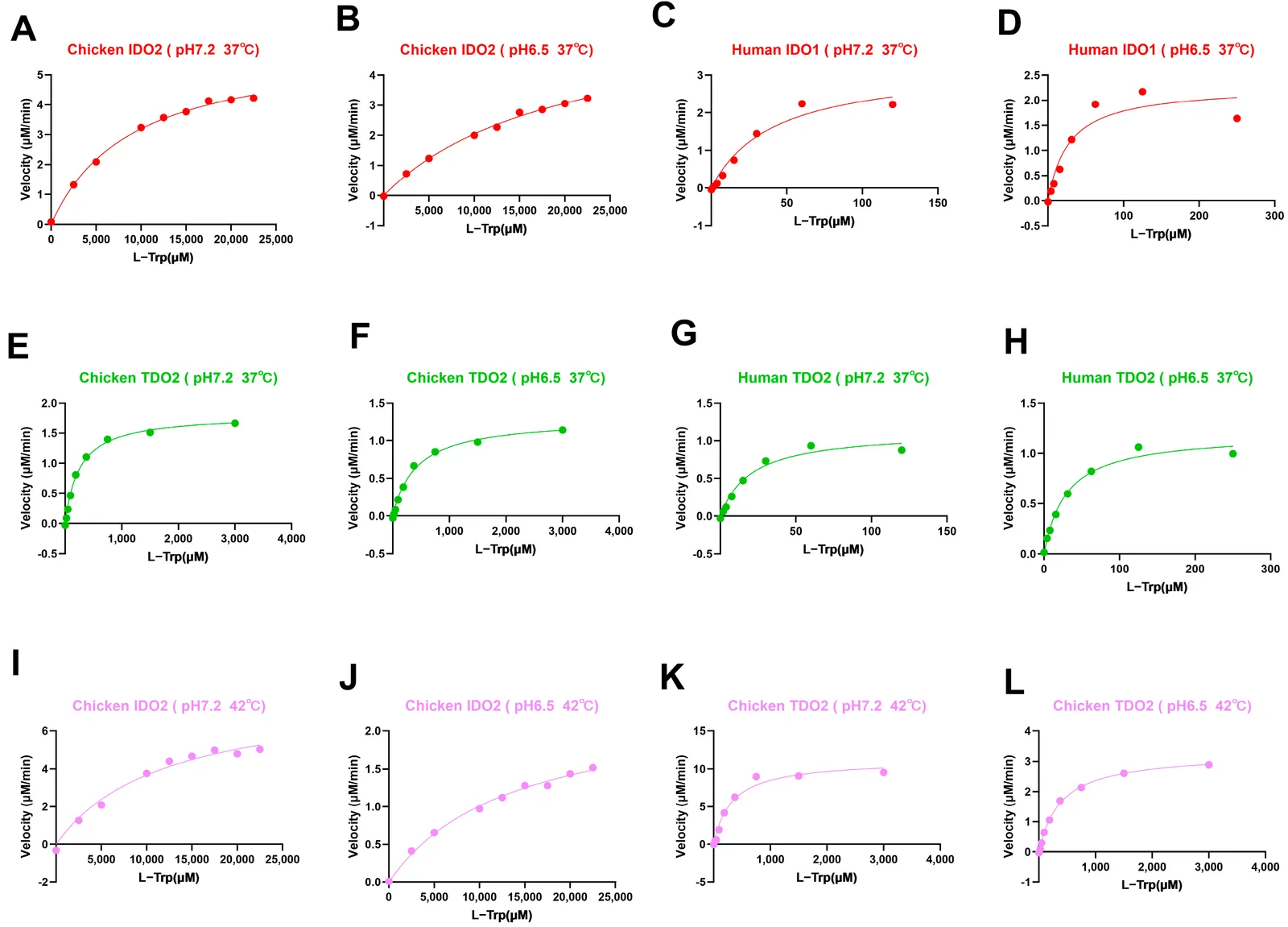
Michaelis-Menten curves of chicken IDO2 and TDO2 reacting with the substrate L-Trp at different pH values and temperatures. (**A**) Chicken IDO2 (pH 7.2 and 37 °C). (**B**) Chicken IDO2 (pH 6.5 and 37 °C). (**C**) Human IDO1 (pH 7.2 and 37 °C). (**D**) Human IDO1 (pH 6.5 and 37 °C). (**E**) Chicken TDO2 (pH 7.2 and 37 °C). (**F**) Chicken TDO2 (pH 6.5 and 37 °C). (**G**) Human TDO2 (pH 7.2 and 37 °C). (**H**) Human TDO2 (pH 6.5 and 37 °C). (**I**) Chicken IDO2 (pH 7.2 and 42 °C). (**J**) Chicken IDO2 (pH 6.5 and 42 °C). (**K**) Chicken TDO2 (pH 7.2 and 42 °C). (**L**) Chicken TDO2 (pH 6.5 and 42 °C).

The enzyme parameters (Km, Vmax, and cat) of chicken IDO2 and TDO2 were analyzed at pH 7.2/pH 6.5 and 37 °C/42 °C, respectively. When the temperature was set to 37 °C, the Km value of chicken IDO2 was greatly affected by the pH. Specifically, the Km value of chicken IDO2 was lower at pH 7.2 than at pH 6.5, indicating that the substrate affinity of chicken IDO2 at pH 7.2 was higher, whereas the Km value of human IDO1 was lower at pH 6.5 and 37 °C (Figure 7). As shown in Table 1, other enzymatic parameters of chicken IDO2, such as Vmax and Kcat, were not significantly affected by pH, whereas human IDO1 showed the opposite to chicken IDO2. Human IDO2 did not exhibit any catalytic activity toward L-Trp in this experiment. Similar to chicken IDO2, when the temperature was set to 37 °C, the pH value greatly affected the Km of chicken TDO2 but did not affect Vmax and Kcat. Specifically, Km was lower and substrate affinity was higher at pH 7.2 than at pH 6.5 for chicken TDO2.

When the temperature was set to 42 °C, the Km value of chicken IDO2 was less affected by pH; however, pH had a greater effect on Vmax and Kcat (Figure 7 and Table 1). Specifically, the Vmax and Kcat values for chicken IDO2 at pH 7.2 were both higher than those at pH 6.5. When the temperature was set to 42 °C, facing different pH values, the changes in the enzymatic parameters of chicken TDO2 were like those of chicken IDO2 (Figure 7 and Table 1).

When pH was set to the physiological pH value (7.2) of chickens, the Km value of chicken IDO2 at 37 °C was less than that at 42 °C, and the Km value of chicken TDO2 at 37 °C was also less than that at 42 °C (Table 1). Chicken IDO2/TDO2’s optimal pH (7.2) aligns with mammalian orthologs (pH 7.0–7.4), while the optimal temperature (42 °C) reflects avian body temperature (39–42 °C), highlighting species-specific enzymatic adaptations.

In this experiment, the catalytic activities of two proteins, IDO2 and TDO2, from chickens on three substrates: 5-OH-Trp, D-Trp, and tryptamine were measured at 42 °C. The maximum concentrations of 5-OH-Trp, D-Trp, and tryptamine were 18.9, 22.5, and 25.0 mM, respectively. All substances reached their maximum solubility in the enzyme reaction system under the experimental conditions. However, chicken IDO2 and TDO2 did not exhibit catalytic activity toward these three substrates, and no products were produced.

**Figure 8:**
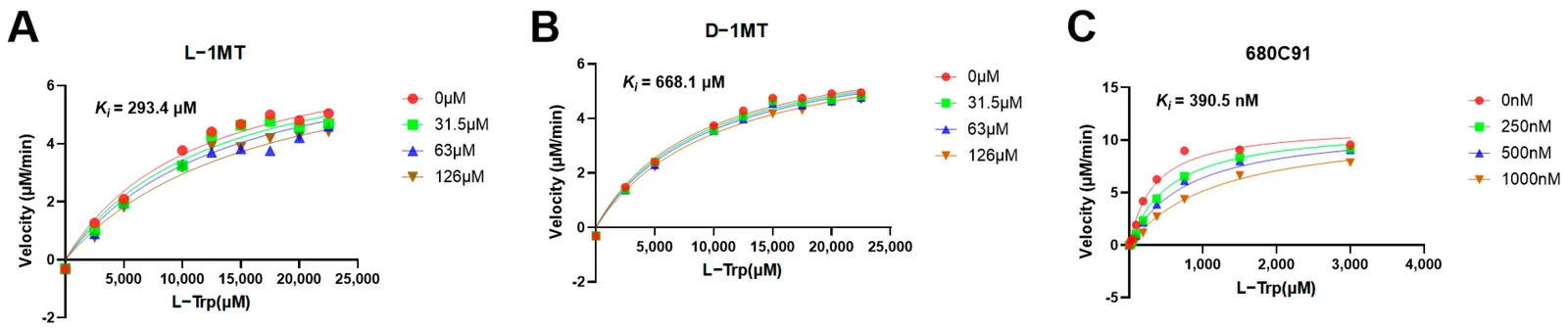
Inhibitory effects of the inhibitors on chicken IDO2 and TDO2. (**A**) The inhibitory effect of L-1MT on chicken IDO2 activity. (**B**) Inhibitory effect of L-1MT on chicken IDO2. (**C**) Inhibitory effect of 680C91 on chicken TDO2 activity. Ki is the inhibition constant. The reaction conditions were as follows: the substrates were all L-Trp, the pH was 7.2, and the temperature was 42 °C.

Chicken IDO2 Ki was assayed using 1-methyl-L-tryptophan (L-1MT) and 1-methyl-D-tryptophan (D-1MT) as inhibitors, and chicken TDO2 Ki was assayed using 680C91 (a commercial human TDO2 inhibitor). This experiment was conducted at pH 7.2 and 42 °C, and the results are shown in Figure 8. The Ki of L-1MT for chicken IDO2 was 293.4 μM, whereas that of D-1MT was 668.1 μM. For chicken TDO2, with the Ki value of 680C91 was 390.5 nM (Figure 8). The smaller the Ki value, the better the inhibitory effect. All three inhibitors inhibited the expression of the corresponding proteins.

### 3.6. The Effects of Chicken IDO2 and TDO2 Overexpression or Knockdown in LMH Cells

**Figure 9:**
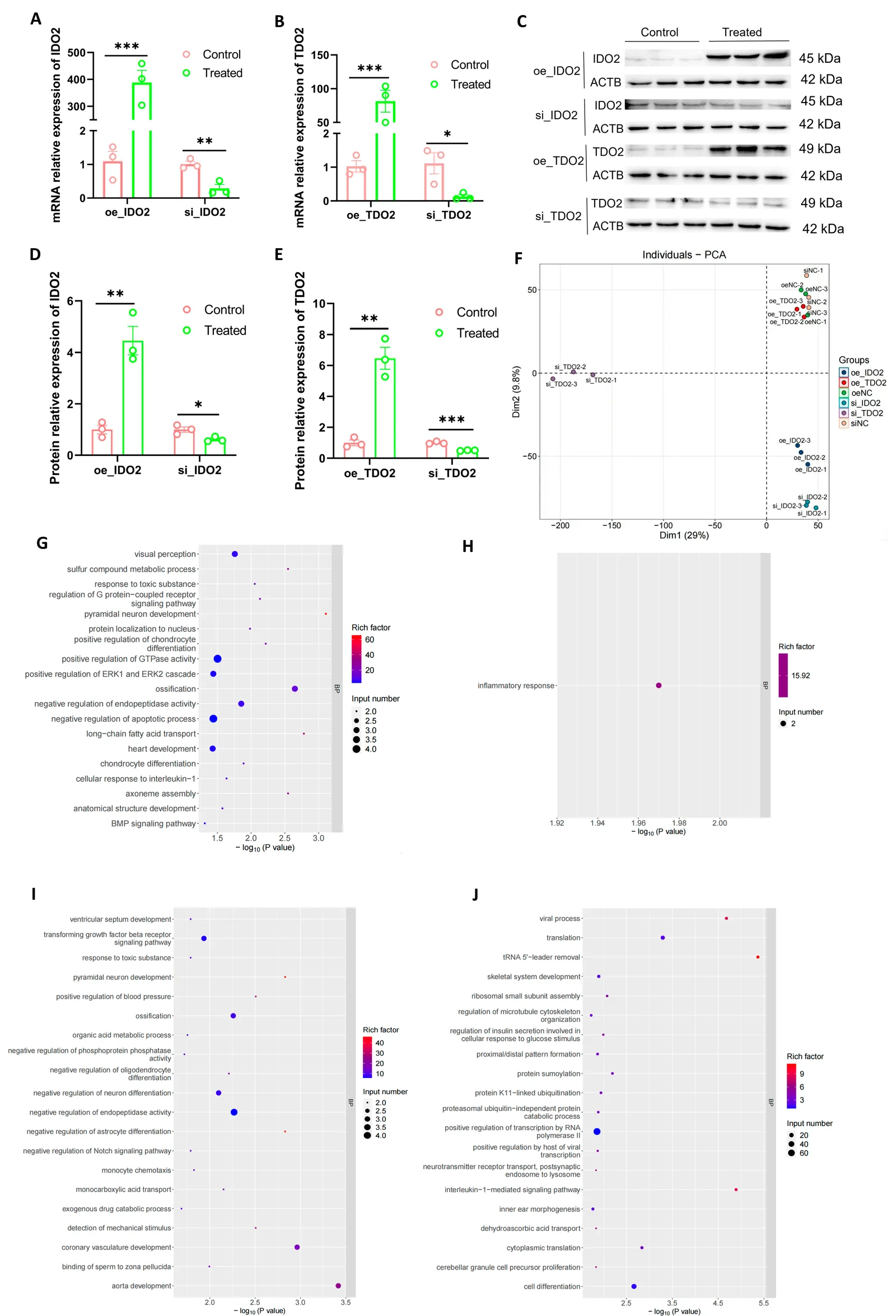
GO enrichment of differentially expressed genes in LMH cells after IDO2 and TDO2 overexpression and knockdown. (**A**) Relative expression of the chicken IDO2 gene at 24 h post-transfection. (**B**) Relative expression of chicken TDO2 gene at 24 h post-transfection. (**C**) Protein expression levels of chicken IDO2 and TDO2 at 36 h post-transfection were detected by Western blot analysis. (**D**) Quantitative analysis of chicken IDO2 protein expression. (**E**) Quantitative analysis of the chicken TDO2 protein expression levels. (**F**) Principal component analysis (PCA) plot of transcriptome sequencing results for each sample group. (**G**) GO enrichment of differentially expressed genes between the IDO2 overexpression group (oe_IDO2) and the control group (oeNC). (**H**) GO enrichment of differentially expressed genes between the TDO2 overexpression group (oe_TDO2) and the control group (oeNC). (**I**) GO enrichment of differentially expressed genes between the IDO2 knockdown group (si_IDO2) and the control group (siNC). (**J**) GO enrichment of differentially expressed genes between the TDO2 knockdown group (si_TDO2) and the control group (siNC). The X-axis of A, B, D, and E represents different experimental objectives. For detailed descriptions of the Control and Treated groups, see Section 2.14. * *p* < 0.05, ** *p* < 0.01 and *** *p* < 0.001 (*n* = 3 per group).

After overexpression and knockdown of chicken IDO2 and TDO2 genes in LMH cells, changes in gene and protein expression were detected, followed by transcriptome analysis. The results are presented in Figure 9. After 24 h of transfection, compared to controls, IDO2 and TDO2 genes showed significant overexpression or knockdown effects (Figure 9A,B), and after 36 h, IDO2 and TDO2 proteins demonstrated significant overexpression and knockdown effects (Figure 9C–E). Principal component analysis of the transcriptome data showed well-clustered replicates for each group, with the si_TDO group far from the others (Figure 9F). After IDO2 overexpression in LMH cells, GO enrichment analysis of differentially expressed genes revealed pyramidal neuron development, long-chain fatty acid transport, and cellular response to interleukin-1 (Figure 9G). After TDO2 overexpression, GO enrichment was only observed in the inflammatory response (Figure 9H). When IDO2 expression was inhibited, GO enrichment involved aorta development, coronary vasculature development, and regulation of astrocyte differentiation (Figure 9I). When TDO2 was knocked down, GO analysis showed genes enriched in biological processes, including tRNA 5’-leader removal, viral process, and interleukin-1-mediated signaling pathway (Figure 9J). Overall, chicken IDO2 and TDO2 may play roles in the interleukin-1-related inflammatory response. However, because the IDO2 knockdown experiment was performed with a single siRNA sequence, the conclusions based on the IDO2 knockdown should be interpreted with appropriate caution (see Section 4).

### 3.7. Changes in Total Enzyme Activity of Chicken IDO2 and TDO2 Under Ex Vivo and In Vitro Inflammatory Conditions

**Figure 10:**
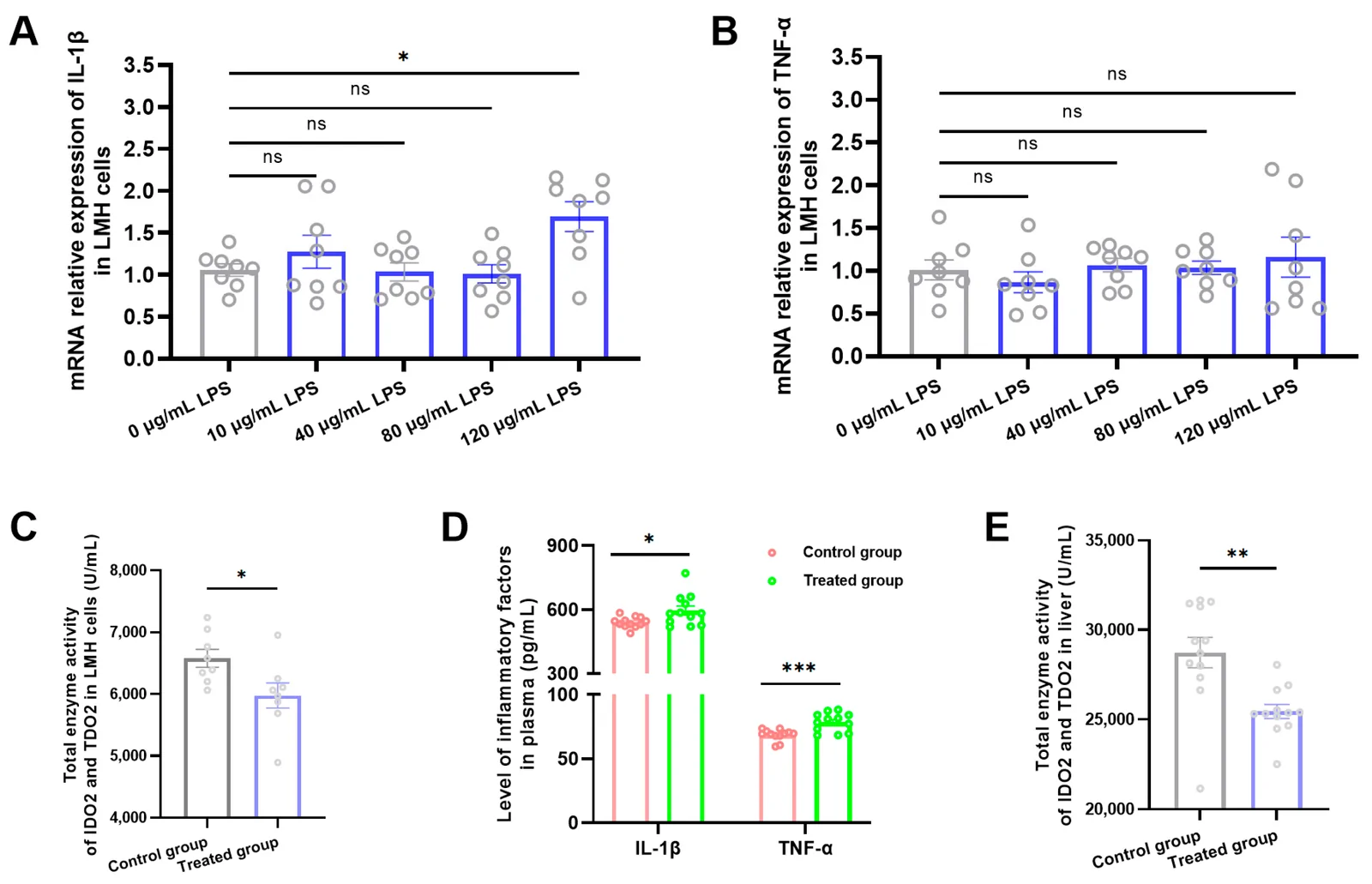
Changes in total enzyme activity of chicken IDO2 and TDO2 under inflammatory conditions. (**A**) Changes in IL-1β mRNA relative expression in LMH cells treated with different concentrations of LPS for 24 h (*n* = 8 per group). (**B**) Changes in TNF-α mRNA relative expression in LMH cells treated with different concentrations of LPS for 24 h (*n* = 8 per group). (**C**) Changes in the total enzyme activity of IDO2 and TDO2 in LMH cells treated with 120 μg/mL LPS for 24 h (*n* = 8 per group). (**D**) Changes in IL-1β and TNF-α levels in the blood of APEC-infected and uninfected chickens (*n* = 12 per group). (**E**) Changes in the total enzyme activities of IDO2 and TDO2 in the livers of APEC-infected and uninfected chickens (*n* = 12 per group). * *p* < 0.05, ** *p* < 0.01, and *** *p* < 0.001; ns, no significant difference.

RT-PCR results showed that after treatment with 120 μg/mL of LPS for 24 h, IL-1β levels in LMH cells showed a significant upward trend compared with the control group (*p* < 0.05), indicating that an inflammatory response was successfully induced in LMH cells (Figure 10A). Thecombined total dioxygenase activities of IDO2 and TDO2 in the inflammatory group of LMH cells were significantly lower than those in the control group (Figure 10C). APEC infected chicks showed clinical signs, such as lethargy, reduced feed intake, and diarrhea et al. APEC infection-induced systemic inflammation was shown in Supplementary Figure S6. After the APEC challenge, IL-1β and TNF-α levels in the blood of infected chickens were significantly upregulated, suggesting that the chickens had an inflammatory response (Figure 10D). Compared to the control group, the combined total dioxygenase activities of IDO2 and TDO2 in the livers of APEC-infected chickens were significantly decreased (Figure 10E).

## 4. Discussion

This study investigated the gene and protein expression, enzymatic activity, and kinetic parameters of chicken IDO2 and TDO2 in Trp metabolism. It also explored their other biological roles, helping to fill research gaps and offering insights for poultry production and immune regulation.

Previous reports show that Trp deficiency in poultry reduces growth and causes weight loss [26]. In our experiment, the control group received the NRC-recommended Trp level (0.2%). When Trp was increased to 0.3%, 0.4%, or 0.5% (groups G1–G3), weight gain slowed, and immune organ indices followed the same trend. Although the reduced weight gain was associated with higher Trp supplementation, the mechanistic interpretation—for example, that energy may be redirected from growth to Trp metabolism and that lipid anabolism may be suppressed via PPAR downregulation [27]—remains speculative, because direct measurements of energy expenditure or energy partitioning were not performed; we therefore present this as a possible mechanism rather than a demonstrated one. However, higher Trp levels (0.3–0.5%) have been shown to benefit broilers under high-density housing [28] or during immune challenges [29]. These differences may stem from variations in feeding conditions, health status, or breed. Our study used healthy Partridge Shank chickens raised under free-range conditions.

Liver transcriptome data help explain these findings. Compared to the control, added dietary Trp upregulated Trp metabolism pathways and affected glycolysis. Blood glucose tests showed that 0.5% Trp significantly lowered blood glucose, indicating altered glucose metabolism [30]. Similar results were seen in pigs: excess Trp (0.78%) caused glucose metabolism disorders [27].

The liver transcriptome also revealed downregulation of the PPAR signaling pathway, which affects lipid metabolism. Liver analysis confirmed a significant drop in lipid droplet content. Interestingly, in obese rat models, Trp intake helped restore normal body weight [31]. This suggests Trp plays a role in regulating energy metabolism in poultry—similar to its function in mammals.

Trp enters the chicken intestine via the mouth, is absorbed by intestinal epithelial cells, and then enters the bloodstream. This study found no major changes in duodenal Trp levels. However, its decomposition products, including L-kynurenine, serotonin, indole-3-lactic acid, and indole-3-carbaldehyde, were significantly increased. This may be attributable to host enzymes and, possibly, to gut microbial metabolism [32,33], although gut microbiota and microbial enzyme activities were not directly measured in this study; therefore, this explanation should be regarded as a hypothesis requiring further investigation.

Blood Trp levels remained stable across most groups, except in the 0.5% group, where they rose significantly. This suggests chickens maintain Trp homeostasis in blood—similar to humans [34]. Measured levels ranged from 99.9 to 196.7 μmol/L (average: 146 μmol/L), close to Birkl’s reported value (105.69 ± 9.20 μmol/L) [17].

Trp decomposition products like L-kynurenine, N-formylkynurenine, and 3-indolelyoxylic acid also rose in blood—but not in a dose-dependent way—highlighting the complexity of Trp metabolism.

Extra dietary Trp markedly increased the combined total IDO2/TDO2 enzyme activity in the liver. At the transcript level, IDO2, TDO2, and KYNU were identified as differentially expressed only under the more permissive threshold (|Fold Change| > 1.4, *p* < 0.05), and qPCR con-firmed the same direction of change; however, the magnitude of these transcriptional changes was modest (|Fold Change| < 2), and at the protein level no significant changes in hepatic IDO2 or TDO2 abundance were detected. Thus, the substantial increase in combined enzyme activity was disproportionately larger than the modest transcriptional changes and occurred without detectable protein-level changes, consistent with post-translational regulation of enzyme activity—a phenomenon also observed for enzymes such as glycogen phosphorylase and isocitrate dehydrogenase [5,35].

We examined the spatial expression of IDO2 and TDO2 in chickens. Like in mammals, chicken IDO2 is found in most organs [36]. Chicken TDO2 is mainly in the liver and thymus—unlike in mammals, where it’s only reported in the liver [37].

Molecular docking predicted similar binding scores between chicken IDO2/TDO2 monomers and L-Trp. We hypothesize that, because TDO2 forms a tetramer, its overall bind-ing to Trp may be stronger than that of IDO2; however, this inference could not be directly verified because the docking software was unable to calculate scores for the TDO2 tetramer.

For enzyme assays, we purified chicken IDO2 and TDO2 using a GST tag, then removed the tag via on-column enzymatic cleavage. Human IDO1, IDO2, and TDO2 were purified the same way for fair comparison. We tested how temperature, pH, substrates, and inhibitors affect chicken IDO2 and TDO2 kinetics. Under our conditions, human IDO2 showed no activity toward L-Trp. Human IDO1 had a Km of 27.77 μM at 37 °C and pH 6.5, which matched earlier studies [21,23]. Chicken IDO2 worked best at pH 7.2 and 37 °C. At 42 °C (chickens’ normal body temperature), its catalytic efficiency (Kcat/Km)) remained similar to 37 °C. The optimal pH for the chicken TDO2 enzyme reaction was 7.2, and the optimal temperature was 42 °C. Chickens have a higher body temperature than mammals, which is most suitable for the enzymatic reactions of chicken IDO2 and TDO2, and pH 7.2 is optimal for these reactions.

Human IDO1 and TDO2 are more efficient than their chicken counterparts, which may explain why chicken blood has higher Trp levels than human blood [17]. Chicken TDO2 was about 10 times more efficient than IDO2, confirming its dominant role in Trp decomposition. Under identical assay conditions, human IDO2 showed no detectable catalytic activity, whereas chicken IDO2 showed measurable activity; because no activity was detected for human IDO2, a quantitative comparison of catalytic efficiency between the two orthologs was not feasible. Neither chicken IDO2 nor TDO2 acted on 5-OH-Trp, D-Trp, or tryptamine. Human inhibitors also affect chicken IDO2 and TDO2, with L-1MT showing stronger inhibitory effects than D-1MT on chicken IDO2 [38,39,40].

Enzyme activity correlated with circulating Trp levels. Chicken TDO2 has high affinity for L-Trp (Km = 0.25–0.42 mM) and is mainly expressed in the liver, the principal site of systemic Trp catabolism; this combination of high substrate affinity and liver-restricted expression suggests that TDO2 likely plays a dominant role in whole-body Trp balance, although this is an inference from in vitro kinetics and tissue distribution rather than direct in vivo flux measurement. In contrast, IDO2 (Km = 8.9–19.9 mM) is widespread but less active, suggesting a comparatively minor contribution to systemic Trp homeostasis. Chicken blood Trp (105.69 ± 9.20 μmol/L) is higher than in humans (67.6 ± 4.9 μmol/L) [17,41]. This may be because human IDO1 and TDO2 have much higher affinity for L-Trp and enzyme activities than chicken IDO2 and TDO2.

To investigate whether chicken IDO2 and TDO2 have functions related to inflammation, like mammal, we overexpressed and knocked down these genes in LMH cells. Transcriptome analysis linked them to inflammatory response pathways. Total enzyme activity of chicken IDO2 and TDO2 dropped significantly under ex vivo and in vitro inflammatory conditions. In mammals, inflammation ususlly boosts IDO1 activity [42]. During viral infections, colitis, or LPS exposure, IDO1 expression and activity rise [43,44,45]. This is thought to suppress excessive T-cell activation by lowering local Trp and producing immunosuppressive kynurenine [46]. However, chickens lack IDO1 and only have IDO2. Current evidence suggests IDO2’s role in inflammation is more complex. Its activity isn’t simply up- or down-regulated. In fact, human IDO2 often has low or no activity due to common genetic variants and evidence suggests that IDO2 acts through non-enzymatic mechanisms [47,48]. IDO2 has been linked to pro-inflammatory effects in autoimmune and skin hypersensitivity models [49,50]. For TDO2, most inflammation studies only measured mRNA or protein, not actual enzyme activity [51]. Since TDO2 is mainly liver-based in both birds and mammals, but most mammalian inflammation studies focus on immune cells or inflamed tissues [52], it’s unclear if tissue-specific regulation exists. Notably, unlike mammalian IDO1 (upregulated in inflammation to deplete Trp) [38], chicken IDO2/TDO2 activity decreases, which could reduce kynurenine production, a proinflammatory metabolite in birds [17]. This may represent an avian-specific negative feedback mechanism, consistent with birds’ reliance on innate immunity [53]. Our results confirm that chicken IDO2 and TDO2 are involved in inflammation. However, more work is needed to clarify whether they act together or separately and how exactly they are regulated. Our findings have practical implications for poultry production: (1) Maintaining dietary Trp at the NRC-recommended 0.2% avoids growth suppression; (2) IDO2/TDO2 inhibitors (e.g., L-1MT, 680C91) could mitigate excessive inflammation in APEC infections, improving disease resistance.

Several limitations of this study should be acknowledged. First, weekly body weights were measured repeatedly on the same birds and were analyzed by between-group comparisons at each individual time point without explicitly modeling within-bird correlation across weeks; a mixed-effects model could be considered in future studies to model within-bird trajectories over time. Second, it should be noted that the IDO2 knockdown experiments relied on a single siRNA sequence, because only one siRNA for chicken IDO2 met the design principles. Knockdown was confirmed at both the mRNA (RT-qPCR) and protein (Western blot) levels; nevertheless, because a second independent IDO2 siRNA and a rescue experiment were not performed, the possibility of off-target effects cannot be formally excluded. The conclusions based on the IDO2 knockdown experiment should therefore be interpreted with appropriate caution, and independent validation using additional siRNAs or a rescue approach will be required in future studies. Third, an independent cell-viability/cytotoxicity assay (e.g., CCK-8, MTT, or LDH-release assay) was not performed at the tested LPS concentrations. Although the upregulation of IL-1β and TNF-α indicates the activation of inflammatory signaling, a contribution of nonspecific LPS-induced cytotoxicity to the observed changes cannot be formally excluded. Future studies should include an independent cell-viability assessment to fully distinguish a specific inflammatory response from nonspecific cellular injury.

## 5. Conclusions

Chicken IDO2 and TDO2 display distinct biochemical catalytic properties adapted to avian physiological conditions. TDO2, which is mainly expressed in the liver and shows high substrate affinity, is the dominant enzyme for systemic tryptophan catabolism and participates in the hepatic response to nutritional and inflammatory stimuli, whereas IDO2, which is widely distributed but has much lower catalytic activity, is likely to exert tissue-specific or regulatory rather than quantitatively dominant functions. These distinct tissue distributions, catalytic efficiencies, and inflammatory response patterns, which differ from mammalian orthologs, define the functional organization of the kynurenine pathway in IDO1-deficient poultry species. These data expand our understanding of avian immunometabolism and provide a biochemical basis for targeted poultry nutritional regulation and disease intervention.

## Figures and Tables

**Table 1:** The enzymatic parameters of chicken IDO2 and TDO2 reacting with the substrate L-Trp under different pH values and temperatures were determined.

Protein	pH	Temperature (°C)	*K_m_* (μM)	*V_max_* (μM ·min^−1^)	*K_cat_* (s^−1^)	*K_cat_*/*K_m_*
Chicken IDO2	7.2	37	8936	6.05	901.30	0.10
Chicken IDO2	6.5	37	19940	6.12	912.49	0.05
Human IDO1	7.2	37	42	3.26	8217.32	196.68
Human IDO1	6.5	37	28	2.28	5741.80	206.76
Chicken TDO2	7.2	37	252	1.81	297.3	1.18
Chicken TDO2	6.5	37	419	1.30	212.92	0.51
Human TDO2	7.2	37	20	1.13	3003.14	147.87
Human TDO2	6.5	37	30	1.20	3184.02	107.39
Chicken IDO2	7.2	42	11918	8.06	1202.34	0.10
Chicken IDO2	6.5	42	13610	2.38	355.45	0.03
Chicken TDO2	7.2	42	320	11.21	1840.30	5.75
Chicken TDO2	6.5	42	384	3.27	537.31	1.40

## Data Availability

Raw transcriptome sequencing datasets were deposited in the Genome Sequence Archive (GSA, accession CRA025867, CRA030411). Metabolomics raw data were uploaded to OMIX repository (OMIX014864). All supporting raw data are available from the corresponding author upon reasonable formal request.

## Supplementary Materials

The following supporting information can be downloaded at: https://www.mdpi.com/article/10.3390/ani16182874/s1, Figure S1. Structures of the three inhibitors. Figure S2. Organ distribution of chicken IDO2 and TDO2. Figure S3. Effect of L-tryptophan on mRNA expression of six genes in chicken liver. Figure S4. Western blot verification of recombinant protein. Figure S5. Protein purification of IDO1, IDO2, and TDO2. Figure S6. APEC infection-induced systemic inflammation in silkie. Table S1. Composition and nutrient levels of the basal diet. Table S2. Primers used for PCR. Table S3. Primers used for qPCR. Table S4. Antibodies used in WB. Table S5. Homologous arm Primers for plasmid construction. Table S6. The siRNA sequence for chicken IDO2 and TDO2.

## Abbreviations

IDO2	Indoleamine 2,3-dioxygenase 2
TDO2	Tryptophan 2,3-dioxygenase
IDO1	Indoleamine 2,3-dioxygenase 1
KP	Kynurenine pathway
L-1MT	1-Methyl-L-tryptophan
D-1MT	1-Methyl-D-tryptophan
APEC	Avian pathogenic Escherichia coli
LPS	Lipopolysaccharide
qPCR	Quantitative PCR
LC-MS/MS	Liquid chromatography-tandem mass spectrometry
DGEs	Differentially expressed genes
GO	Gene Ontology
KEGG	Kyoto Encyclopedia of Genes and Genomes
PCA	Principal component analysis
VIP	Variable importance in the projection
FDR	False discovery rate
IPTG	Isopropyl β-D-1-thiogalactopyranoside
PBS	Phosphate-buffered saline
OCT	Optimal cutting temperature
GAPDH	Glyceraldehyde-3-phosphate dehydrogenase
ACTB	β-actin
ECL	Enhanced chemiluminescence
CFU	Colony-forming unit
NRC	National Research Council
LMH	Leghorn Male Hepatoma (chicken liver cell line)
GST	Glutathione S-transferase
SDS-PAGE	Sodium dodecyl sulfate–polyacrylamide gel electrophoresis
