# Supplementary material for: Chicken IDO2 and TDO2: Biochemical Characteristics and Regulatory Roles in Avian Tryptophan Metabolism and Inflammatory Response
Source: Animals (Basel). 2026 Sep 12;16(18):2874. doi: 10.3390/ani16182874 (PMC13603309; doi:10.3390/ani16182874)
Supplement: Supplementary file 1 [file animals-16-02874-s001.zip › animals-4548560-supplementary.pdf]

Supplementary information

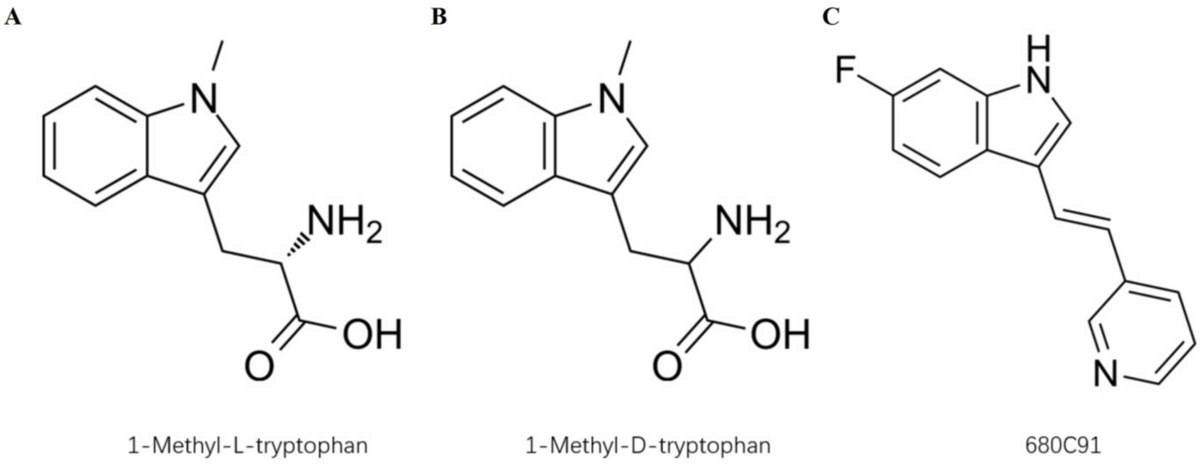

Figure S1. Structures of the three inhibitors. (A) 1-methyl-L-tryptophan (L-1MT), an inhibitor of IDO2. (B) 1-methyl-D-tryptophan (D-1MT), an IDO2 inhibitor c 680C91, an inhibitor of TDO2.

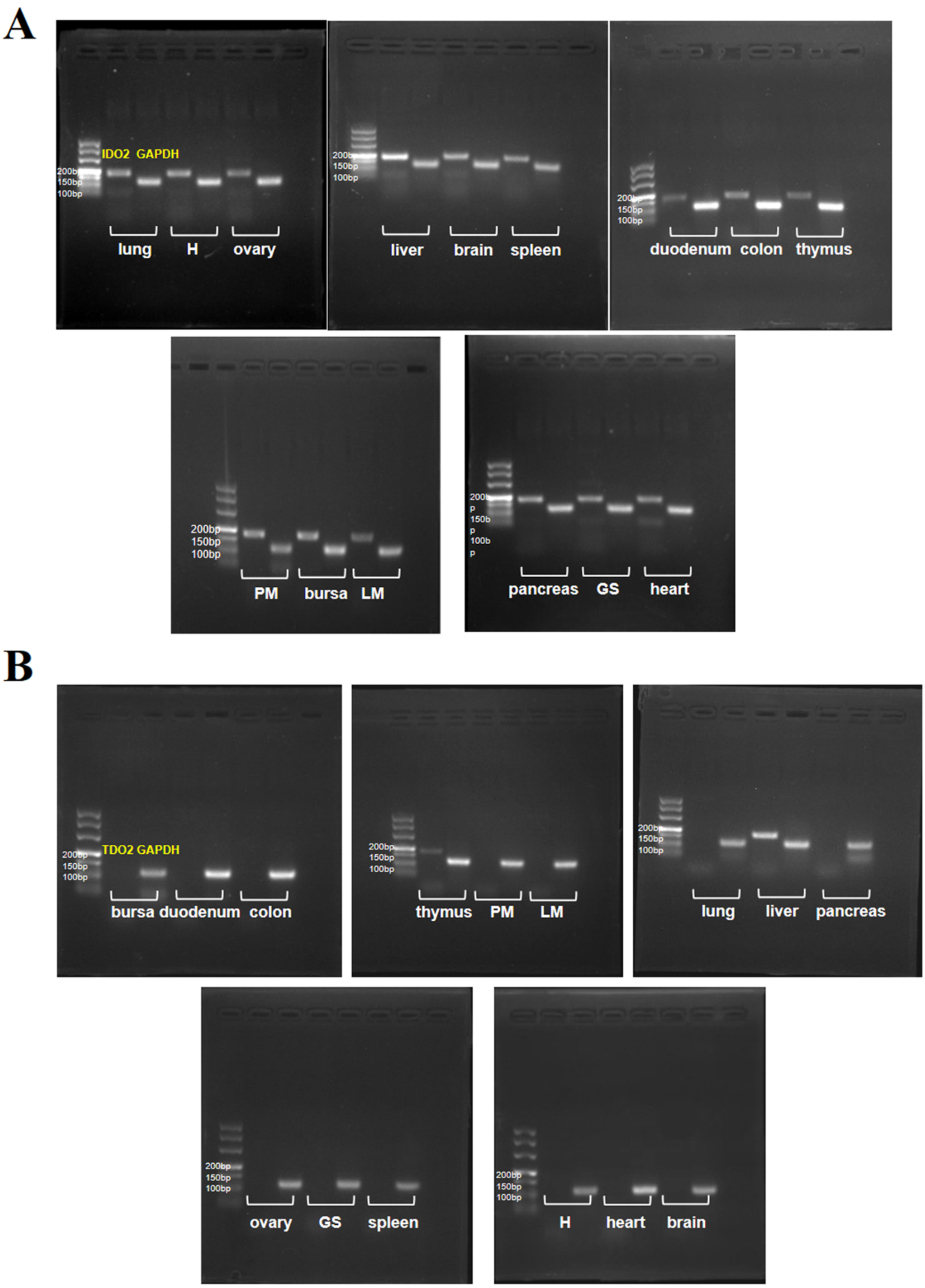

Figure S2. Organ distribution of chicken *IDO2* and *TDO2*. (A) Organ distribution of chicken *IDO2*. (B) Organ distribution of chicken *TDO2*. H represents hypothalamus. PM represents

the pectoral muscle. LM represents the leg muscle. GS, glandular stomach. The leftmost lane of each figure shows the DNA marker. The left lane contains the targeted gene; the right lane holds *GAPDH* (positive control) within this organ's set lanes.

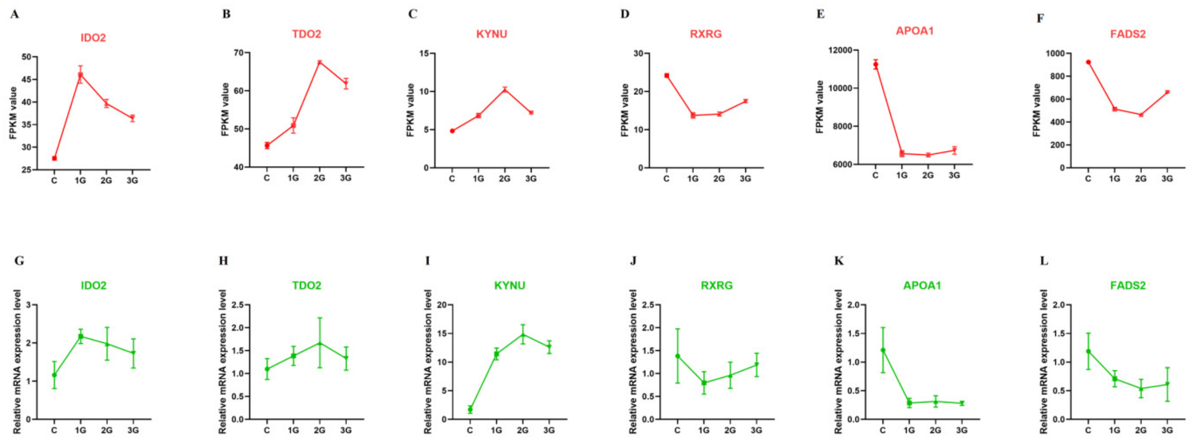

Figure S3. Effect of L-tryptophan on mRNA expression of six genes in chicken liver. The red lines and symbols represent the FPKM values of the transcriptomic data. Green lines and symbols represent the values detected by qPCR. (A) and (G) *IDO2* gene. (B) and (H) *TDO2* gene. (C) and (I) *KYNU* gene. (D) and (J) *RXRG* genes. (E) and (K) *APOA1* genes. (F) and (L) *FADS2* (*n* = 4 in each group).

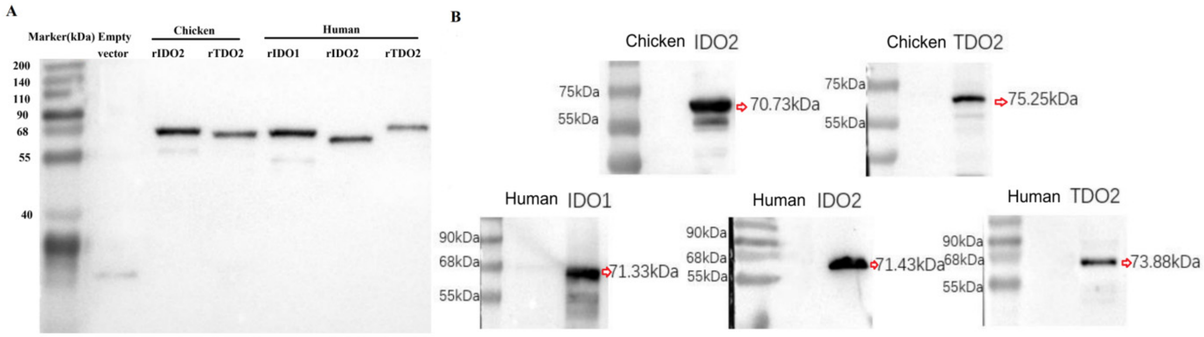

Figure S4. Western blot verification of recombinant protein. (A) GST antibody incubation of recombinant protein. From left to right, the lanes are marker, empty vector, recombinant chicken IDO2, TDO2, recombinant human IDO1, IDO2, and TDO2 proteins. (B) Endogenous antibody incubation with recombinant proteins. The corresponding five figures show the western blot validation of endogenous antibodies against chicken IDO2 and TDO2 and human IDO1, IDO2, and TDO2. The first lane is the marker, the second lane is the empty vector, the third lane is the target fusion protein, and the right side is the corresponding protein molecular weight.

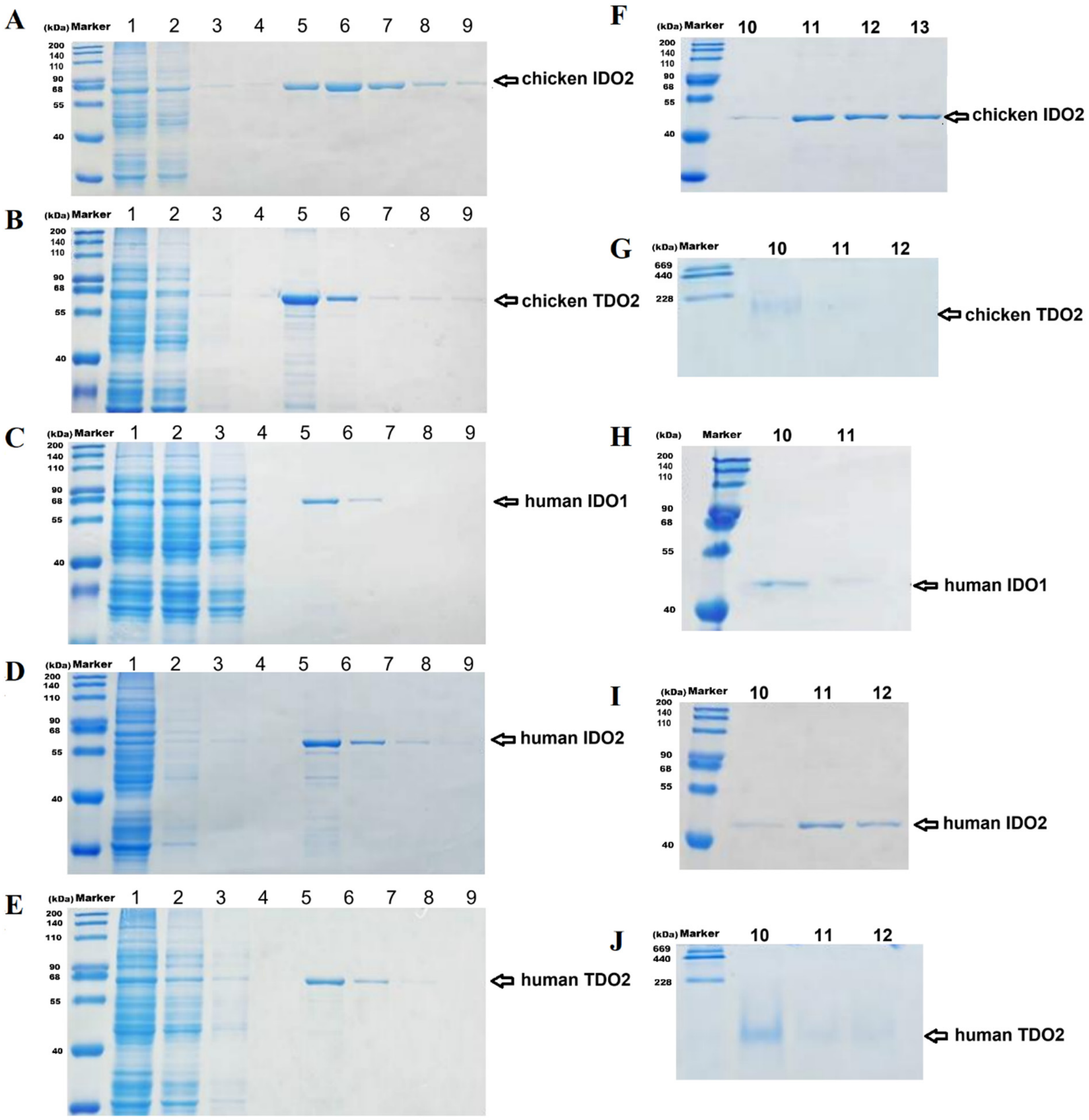

Figure S5. Protein purification of IDO1, IDO2, and TDO2. (A)- (E) Protein purification with GST tag. (F)-(J) Protein purification with the GST tag removed. The black arrow indicates the purified protein. Columns 1 and 2 are flow-through solutions; columns 3 and 4 are wash solutions for PBS; and columns 5, 6, 7, 8, and 9 are elution solutions for reduced glutathione. Columns 10, 11, 12, and 13 are flow-through solutions for thrombin removal after cutting the GST tag. Except for panels (G) and (J), which show native-PAGE (TDO2 is a tetramer), the

rest are SDS-PAGE. The GST label is 26 kDa, and after removing the GST tag, the chicken IDO2 protein molecular weight is 45 kDa, the chicken TDO2 monomer is 49 kDa, and the chicken TDO2 tetramer is 196 kDa. Human IDO1 protein molecular weights of kDa, human IDO2, TDO2 monomer is 48 kDa, and TDO2 tetramer are 45, 45, 48, and 192 kDa, respectively.

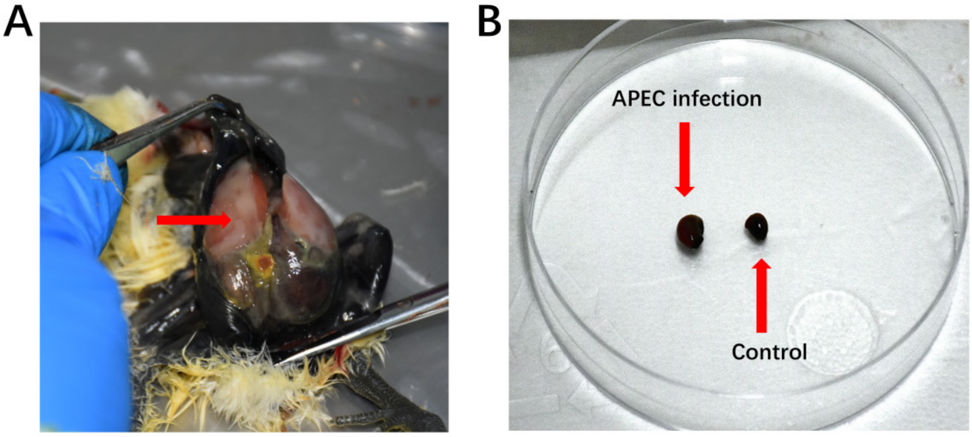

Figure S6. APEC infection-induced systemic inflammation in silkie. A indicates the presence of white necrotic spots in the liver. B indicates splenomegaly.

Table S1. Composition and nutrient levels of the basal diet (%, on a dry matter basis)

Items	Content
Ingredients	
Corn	58.0
Soybean meal	22.0
Cotton meal	5.3
Feather powder	1.5
Corn gluten meal	2.0
DDGS Distillers Dried Grains with Solubles	5.5
CaHPO4	1.2
Stone powder	1.5
Animal oil	1.0
Premix[1]	2.0
Total	100.0
Nutrient levels	
Crude protein(CP)	25.78
Methionine	0.54
Lysine	1.68
Tryptophan	0.23
Calcium (g/kg)	14
Total phosphorus	0.70
Metabolism energy (ME) (MJ/kg)	13.40

[1] The premix was incorporated at 2% of the basal diet and provided the following per kilogram of complete diet: vitamin A (as retinyl acetate), 12,500 IU; cholecalciferol (vitamin D_3), 4,000 IU; DL-α-tocopheryl acetate (vitamin E), 30 IU; menadione sodium bisulfite (vitamin K_3), 2.5 mg; thiamine mononitrate (vitamin B_1), 2.5 mg; riboflavin (vitamin B_2), 7.5 mg; pyridoxine hydrochloride (vitamin B_6), 3.5 mg; cyanocobalamin (vitamin B_{12}), 0.025 mg; niacin, 35 mg; D-calcium pantothenate, 12 mg; folic acid, 1.0 mg; D-biotin, 0.15 mg; choline chloride (50% purity), 1,000 mg; iron (from ferrous sulfate heptahydrate), 80 mg; copper (from copper sulfate pentahydrate), 10 mg; zinc (from zinc sulfate monohydrate), 75 mg; manganese (from manganese sulfate monohydrate), 100 mg; iodine (from potassium iodide), 0.6 mg; selenium (from sodium selenite), 0.3 mg. Corn starch was used as the carrier for the premix.

Table S2. Primers used for PCR

Gene Symbol	Accession Number	Primer Sequence	Size (bp)
IDO2	>XM_040689374.2	5’-GCCACCTTCCTACAGCGTT<br>5’-TGGTGAGAAAGCTGAGCACC	167
TDO2	>ENSGALT00000154649.1	5’-CATGTCTGCATGGTGCACAG<br>5’-GGTATCCACTGTCTTGGCACT	155
GAPDH	>NM 204305.2	5’-AGAACATCATCCCAGCGTCC<br>5’-ACAGAGACATTGGGGGTTGG	115

Table S3. Primers used for qPCR

Gene Symbol	Accession Number	Primer Sequence	Size (bp)
ACTB	>NM_205518.2	F: GTTACTCCCACAGCCAGCCAT<br>R: CGTAGCTGTCTTTCTGGCCC	179bp
IDO2	>XM_040689374.2	F: GCCACCTTCCTACAGCGTT<br>R: TGGTGAGAAAGCTGAGCACC	167bp
TDO2	>ENSGALT00000154649.1	F: CATGTCTGCATGGTGCACAG<br>R: GGTATCCACTGTCTTGGCACT	155bp
KYNU	>XM_046944157.1	F: GTTTGGGCAGAGTAGGTGTGT<br>R:<br>CAGGTCATTCAATTTCACCTTTGT	105bp
RXRG	>NM_205294.2	F: CTCTTCAACCCAGACGCCAA<br>R: TGCTTCGTGTAGGCTTCCAG	95bp
APOA1	>NM_205525.5	F: TGGGCAAACAGCTTGACCTGAA<br>R: GAAGGGCCGGATCTTCTCCT	188bp
FADS2	>NM_001160428.3	F: CAATGCCACGGCACAATTTC<br>R: GTGACCTGTTCCCTCCCCAT	199bp
IL-1β	>NM_204524.2	F: GTGAGGCTCAACATTGCGCTG<br>R: CCGCTCATCACACACGACATGT	122bp
TNF-α	>NM_204267.2	F:CCGCCCAGTTCAGATGAGTT<br>R:GCAACAACCAGCTATGCACC	130bp

Table S4. Antibodies used in WB.

**Antibody (Item No.)**	Specificity	Company
ACTB(AA128)	WB (1:1000)	Beyotime
IDO2(30530-1-AP)	WB (1:1000)	proteintech
TDO2(15880-1-AP)	WB (1:500)	proteintech
IDO1(13268-1-AP)	WB (1:1000)	proteintech
GST tag	WB (1:4000)	Signalway antibody

Table S5. Homologous arm Primers for plasmid construction

Gene Symbol	Accession Number	Vector	Primer Sequence	Size (bp)
Chicken IDO2	XM_040689374.2	pGEX-4T-3	F:5’- ccccgaattcccgggtcgacATGGAGGCTGGGGACGAG<br>R:5’- gatgcggccgctcgagtcgacTCAGGCACTGATCAGCCCC	1260
Chicken TDO2	ENSGALT00000154649.1	pGEX-4T-3	F: 5’-tccccgaattcccgggtcgacATGAGAAAAGAGCACAGCTCAGTG<br>R:5’-gatgcggccgctcgagtcgacTCAGTCAGAGTCATCACTGCTAAAATAG	1305
Human IDO1	NM_005651.4	pGEX-4T-3	F: 5’-tccccgaattcccgggtcgacATGGCACACGCTATGGAAAAC<br>R:5’- gatgcggccgctcgagtcgacTTAACCTTCCTTCAAAAGGGATTT	1254
Chicken IDO2	XM_040689374.2	pcDNA3.1-3xFLAG	F: 5’-gacgataagaagcttggtaccATGGAGGCTGGGGACGAG<br>R:5’- agtggatccgagctcggtaccTCAGGCACTGATCAGCCCC	1260
Chicken TDO2	ENSGALT00000154649.1	pcDNA3.1-3xFLAG	F: 5’-gacgataagaagcttggtaccATGAGAAAAGAGCACAGCTCAGTG<br>R:5’- aacgggccctctagactcgagTCAGTCAGAGTCATCACTGCTAAAATAG	1305

Note: Lowercase letters indicate the vector sequences. The underlined letters indicate the enzyme cutting sites. Uppercase letters indicate target gene sequences.

Table S6. The siRNA sequence for chicken IDO2 and TDO2

siRNA	sequence
si-IDO2	Sense:5'- UGCAGCAGAUGCACGAUUA/dT/dT/<br>Antisense:5'- UAAUCGUGCAUCUGCUGCA/dT/dT/
si-TDO2 - Ⅰ	Sense: 5'-AGCUGUCUUUGAAAGAUGA/dT/dT/<br>Antisense:5'-UCAUCUUUCAAAGACAGCU/dT/dT/
si-TDO2 - Ⅱ	Sense: 5'-GGAGAUUACCUGCAAUUGA/dT/dT/<br>Antisense:5'-UCAAUUGCAGGUAAUCUCC/dT/dT/
si-TDO2 - Ⅲ	Sense: 5'-GAAUUUCAAUGAUUCUGAA/dT/dT/<br>Antisense:5'-UUCAGAAUCAUUGAAAUUC/dT/dT/
si-NC	Sense: 5'-UUCUCCGAACGUGUCACGUTT<br>Antisense:5'-ACGUGACACGUUCGGAGAATT

Note: *IDO2* was designed as an siRNA. *TDO2* was designed using three siRNAs that were added simultaneously during transfection.
